# Naive Pluripotent Stem Cells Derived Directly from Isolated Cells of the Human Inner Cell Mass

**DOI:** 10.1016/j.stemcr.2016.02.005

**Published:** 2016-03-03

**Authors:** Ge Guo, Ferdinand von Meyenn, Fatima Santos, Yaoyao Chen, Wolf Reik, Paul Bertone, Austin Smith, Jennifer Nichols

**Affiliations:** 1 Wellcome Trust – Medical Research Council Stem Cell Institute, University of Cambridge, Tennis Court Road, Cambridge CB2 1QR, UK; 2 Department of Physiology, Development and Neuroscience, University of Cambridge, Downing Street, Cambridge CB2 4BG, UK; 3 Department of Biochemistry, University of Cambridge, Tennis Court Road, Cambridge CB2 1GA, UK; 4 Epigenetics Programme, Babraham Institute, Cambridge CB22 3AT, UK; 5 Wellcome Trust Sanger Institute, Hinxton, Cambridge CB10 1SA, UK

## Abstract

Conventional generation of stem cells from human blastocysts produces a developmentally advanced, or primed, stage of pluripotency. In vitro resetting to a more naive phenotype has been reported. However, whether the reset culture conditions of selective kinase inhibition can enable capture of naive epiblast cells directly from the embryo has not been determined. Here, we show that in these specific conditions individual inner cell mass cells grow into colonies that may then be expanded over multiple passages while retaining a diploid karyotype and naive properties. The cells express hallmark naive pluripotency factors and additionally display features of mitochondrial respiration, global gene expression, and genome-wide hypomethylation distinct from primed cells. They transition through primed pluripotency into somatic lineage differentiation. Collectively these attributes suggest classification as human naive embryonic stem cells. Human counterparts of canonical mouse embryonic stem cells would argue for conservation in the phased progression of pluripotency in mammals.

## Highlights

• Karyotypically normal pluripotent stem cells derived from single human ICM cells
• Expanded lines retain anticipated molecular features of naive embryonic stem cells
• Consistency between ICM-derived and in vitro reset naive human pluripotent stem cells
• Presence of KLF17 protein in human ICM and naive stem cells

## Graphical Abstract

**Figure:**
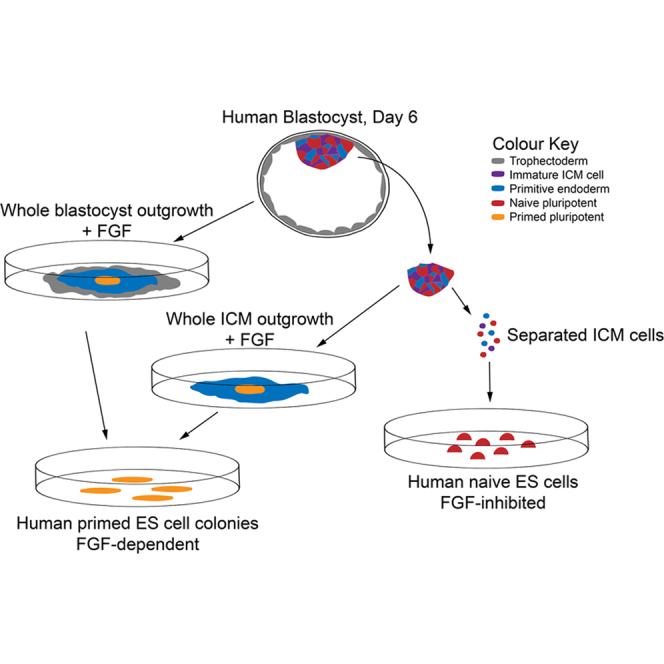

## Teaser

In this paper, Nichols, Smith, and colleagues show that stem cells can be derived from human embryos by dissociation of the inner cell mass into single cells and culture in conditions that suppress further differentiation. Molecular profiling indicates a more naive status than previously observed for human embryonic stem cell lines.

## Introduction

Human pluripotent stem cells (PSCs), whether derived from blastocysts or generated by somatic cell reprogramming, differ substantially from canonical mouse embryonic stem cells (ESCs) and are considered to represent a later phase of epiblast development, termed primed pluripotency (Hackett and Surani, 2014, Nichols and Smith, 2009, Rossant, 2015). Multiple claims of conversion of primed human PSCs into a more naive-like phenotype have been published (reviewed in (Davidson et al., 2015)). These reports are based on a shift in some attribute(s) in response to exogenous reprogramming factors and/or altered culture conditions. Evidence has been lacking, however, for a global state that correlates with mouse ESCs or human naive epiblast (Huang et al., 2014), or for presence of a functional gene regulatory network to sustain naive pluripotency (Boroviak et al., 2015, Dunn et al., 2014, Martello and Smith, 2014).

Two independent studies have described resetting of human PSCs to resemble mouse ESCs following short-term expression of *KLF2* and *NANOG* (Takashima et al., 2014, Theunissen et al., 2014). Reset cells are maintained in medium based on components used for mouse ESCs (Dutta et al., 2011, Ying et al., 2008) comprising titrated inhibition of glycogen synthase kinase-3 and blockade of the mitogen-activated protein kinase (MAPK/Erk) pathway (t2i) with leukemia inhibitory factor (LIF), plus protein kinase C (PKC) inhibition (Takashima et al., 2014). LIF and t2i have also been used to achieve resetting in combination with activin plus inhibitors of BRaf, Src family kinases, and Rho-associated kinase (ROCK) (Theunissen et al., 2014). Reset pluripotent cells are transcriptionally distinct from conventional PSCs and more similar to mouse ESCs and human ICM (Davidson et al., 2015, Huang et al., 2014). They have increased mitochondrial respiratory activity and exhibit global DNA hypomethylation (Takashima et al., 2014), properties consistent with pre-implantation identity. Perhaps most persuasively, reset cells have acquired expression of, and functional dependency on, transcription factors KLF4 and TFCP2L1 constituting part of the core gene regulatory network of naive pluripotency in mouse ESCs (Dunn et al., 2014, Martello et al., 2013, Niwa et al., 2009, Ye et al., 2013) and are expressed in the human ICM but negligible in the primed PSC (Takashima et al., 2014).

In rodents functional equivalence of ESCs with naive epiblast can be demonstrated by blastocyst colonization and extensive multilineage contribution to chimeras. Such an assay is not feasible in human. An alternative indicator of developmental identity is propagation directly from naive epiblast cells, as for derivation of mouse ESCs (Boroviak et al., 2014, Brook and Gardner, 1997, Nichols et al., 2009). In human the standard process for establishing PSC lines from embryos entails explant outgrowth to form an epithelial structure (Pickering et al., 2003), the post-inner cell mass intermediate (PICMI) (O'Leary et al., 2012). This is thought to simulate development of the post-implantation embryonic disk (Van der Jeught et al., 2015), which may explain why derivative cell lines acquire characteristics of primed pluripotency. Naive pluripotency factors such as TFCP2L1 are downregulated during PICMI formation (O'Leary et al., 2012). We elected to test the ability of culture conditions that sustain human naive PSCs after resetting in vitro to support de novo derivation from dissociated human ICMs without PICMI transition.

## Results

Previous human embryo derivations of PSCs have been performed in the presence of fibroblast growth factor (FGF) and/or serum factors, conditions that support developmental progression. We avoided these and adopted the culture regime developed for human reset PSCs (Takashima et al., 2014), comprising serum-free N2B27 medium with LIF and t2i (inhibitors of GSK3 and MAPK/Erk signaling) plus the PKC inhibitor Gö6983. To safeguard viability of precious embryo cells, we added ascorbic acid and ROCK inhibitor (Y-27632), constituting t2iLGöY. Cultures were maintained throughout on fibroblast feeders in 5% O_2_.

**Figure 1:**
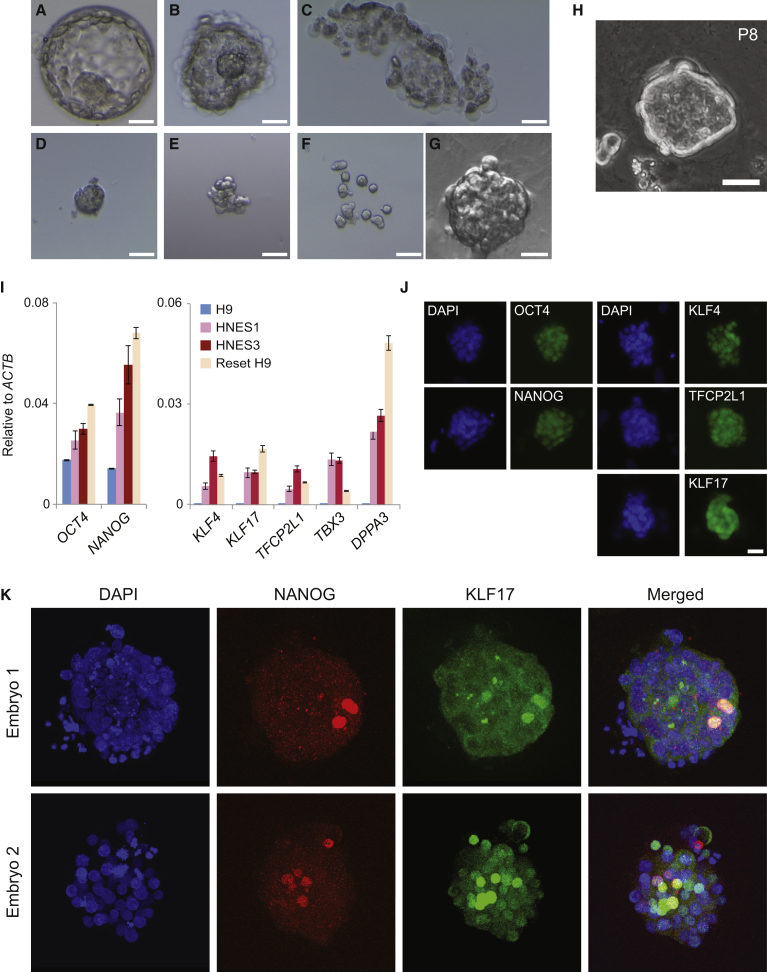
Cell Line Derivation from Dissociated Human Inner Cell Mass Cells (A) Day-6 blastocyst. (B) Trophoblast lysis. (C) Discarded trophoblast. (D) Isolated inner cell mass. (E) Decompacted ICM. (F) Dissociated ICM. (G) Primary stem cell clone grown from a single ICM cell. (H) Colony at passage 8. (I) qRT-PCR for pluripotency markers in HNES cells, conventional human PSCs (H9), and in vitro reset PSCs (Reset H9). Error bars indicate the SD of two independent reactions. (J) Immunofluorescence of pluripotency markers in HNES1 cells. (K) Immunofluorescence of KLF17 and NANOG in D6 human ICM cells. Scale bars: 25 μm.

ICMs were isolated from blastocysts 6 days post-fertilization by immunosurgery (Solter and Knowles, 1975). Following dissociation, single cells or doublets were distributed on feeders in t2iLGöY. Up to half of the plated ICM cells formed compact colonies within 4–5 days (Figures 1A–1G), similar to mouse ESC primary colony formation (Nichols et al., 2009). For each embryo, colonies were manually picked, dissociated, and pooled. Replated cells proliferated (Figures 1H and S1A) and from a total of eight ICMs, four cell lines were established (Table 1) and provisionally termed human naive embryonic stem (HNES) cells.

HNES cells were expanded by passaging every 3–4 days, with ROCK inhibitor and ascorbic acid maintained throughout. HNES cells can be replated and maintained without ROCK inhibitor, albeit at lower efficiency, and propagated without ascorbic acid (Figures S1B–S1D). They can be cryopreserved and thawed with expected recovery efficiency using standard procedures. HNES1 cells exhibit a consistent 46XY karyotype with no abnormalities detected by G-banding (Figure S1E and Table S1), while HNES2 comprised both diploid and tetraploid cells on initial karyotyping but resolved to 46XY after flow sorting (Figures S1E and S1F). HNES3 is a mix of 46XX and cells with chr22 trisomy. HNES4 contains two isochromosomes of chromosome 12. Array comparative genomic hybridization at 200 kb genome-wide resolution confirmed lack of chromosomal abnormalities in HNES1. This line is described below with data from other lines where specified.

HNES cells expressed mRNAs for naive pluripotency markers *KLF4*, *TFCP2L1*, and *DPPA3*, along with elevated *NANOG* transcripts (Figure 1I) as seen in reset cells generated from conventional PSCs (Takashima et al., 2014). Immunostaining confirmed presence of NANOG, KLF4, TFCP2L1, and OCT4 (Figures 1J and S1G). Expression of *ESRRB* and *KLF2* was low in HNES cells, similar to reset cells. Both factors are also expressed at low levels in human and marmoset ICMs, indicating divergence between primates and rodents (Blakeley et al., 2015, Boroviak et al., 2015). Another Kruppel-like factor, KLF17, is observed at the transcript level in primate ICMs (Blakeley et al., 2015, Boroviak et al., 2015) and expressed in reset and HNES cells (Figure 1I). We detected KLF17 protein in HNES cells and human ICMs (Figures 1J and 1K).

**Figure 2:**
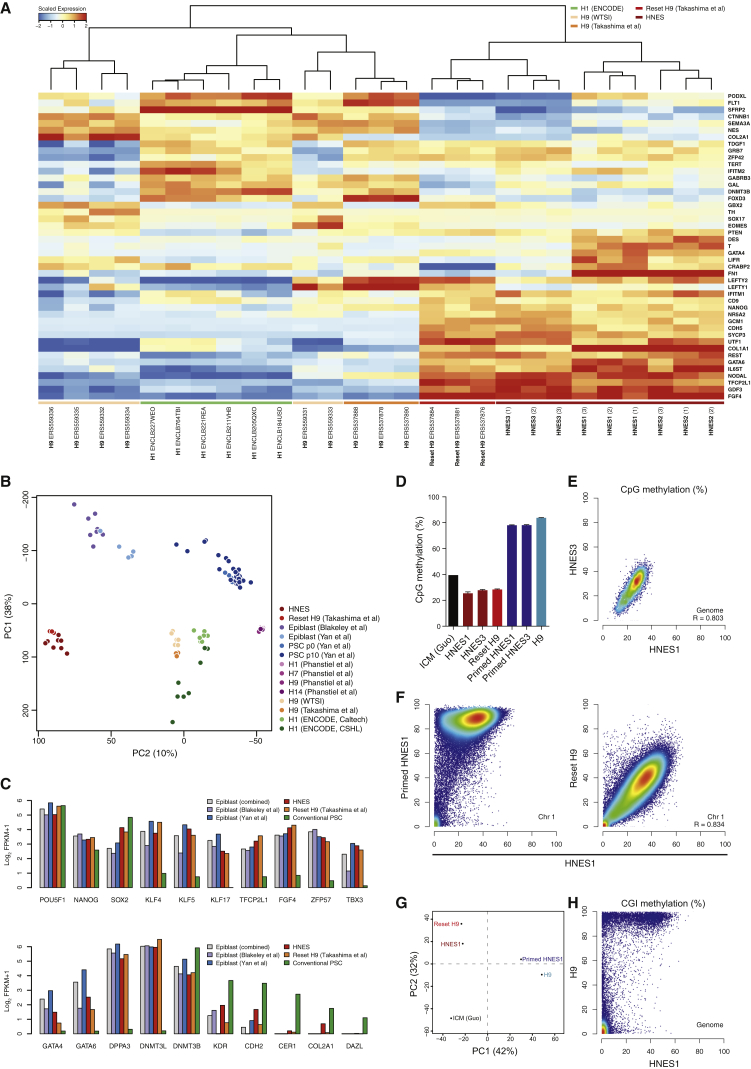
Transcriptome and Methylome Analyses (A) Clustered expression data from HNES cells, and reset and conventional human PSCs for a panel of pluripotency and lineage markers selected by the International Stem Cell Initiative (Adewumi et al., 2007). Displayed are log_2_ FPKM values (fragments per kilobase of exon per million reads mapped) scaled by the mean expression of each gene across samples. Published data are labeled with sample accession codes. (B) PCA of HNES cells, and reset and conventional PSCs with single-cell RNA-seq data from early human ICMs (Blakeley et al., 2015, Yan et al., 2013) and PSC explants. Embryo single-cell samples are those assigned an epiblast identity in the respective studies. (C) Pluripotency and lineage marker expression in human ICM, HNES cells, and reset and conventional PSC lines. (D) Proportion of whole-genome CpG methylation measured by bisulfite sequencing (BS-seq) analysis from three biological replicates. Error bars indicate the SD of three biological replicates. (E) Comparison of global methylation in HNES1 (male) and HNES3 (female) cells by averaging CpG methylation levels over 500-kb windows. (F) Comparisons of CpG methylation in HNES1 cells and primed derivatives, and reset H9 and ICM cells. (G) PCA of mean CpG island methylation. (H) CGI methylation in HNES1 and conventional PSCs.

Whole-transcriptome profiles were obtained by RNA-seq from replicate cultures of HNES1, HNES2, and HNES3. These were compared with reset and conventional human PSC datasets (Takashima et al., 2014) and to a wider panel of H1, H7, H9, and H14 data from the public domain. HNES cells feature a transcriptome distinct from other PSCs and close to the reset state (Figure 2A). They show consistent expression of naive pluripotency factors. Conventional PSCs exhibit wider variation in expression profiles with sporadic activation of naive factors such as *NANOG, ZFP42* (*REX1*), and *TFCP2L1*. HNES cells express a restricted complement of lineage markers compared with conventional PSCs. We performed principal component analysis (PCA), additionally incorporating published data (Blakeley et al., 2015, Yan et al., 2013) on human ICM cells and primary cultures generated by single-cell RNA-seq (Figure 2B). PC1 primarily discriminates between cells profiled by single-cell and bulk RNA-seq methods, suggesting a substantial contribution of global expression variance by sequencing protocol. Numerous transcripts present in conventional RNA-seq datasets register zero read counts in the single-cell libraries, in line with known detection limitations (Kharchenko et al., 2014). Biological replicates of the three HNES cells cluster together and adjacent to reset H9 cells. PC2 places HNES cells in relative proximity to the ICM cells and well separated from other PSCs. The degree of correspondence between HNES and embryo cells appears reasonable, considering the wider variation between samples profiled in the embryo studies, and that early ICM cells analyzed precede naive epiblast. Markers of naive pluripotency and lineage specification diverge between HNES and reset cells versus conventional PSC (Figure 2C).

Primed PSCs rely on anaerobic glycolysis with low mitochondrial respiration capacity (Zhou et al., 2012), whereas reset PSCs have active mitochondria and reduced glucose dependence (Takashima et al., 2014). We evaluated the capacity of HNES cells to form colonies in the presence of the competitive inhibitor of glycolysis, 2-deoxyglucose. Undifferentiated HNES cells readily formed colonies while primed HNES cells generated by passaging in FGF/KSR did not survive (Figure S2A). HNES cells also stained intensely with MitoProbe DiIC1, reflecting mitochondrial membrane potential (Figures S2B and S2C). Extracellular flux analysis indicated that HNES cells exhibit at least 2-fold higher respiratory capacity than primed cells (Figure S2D).

Global DNA hypomethylation is a distinguishing feature of mouse and human ICM cells (Guo et al., 2014, Smith et al., 2012), a property shared with naive ESCs (Ficz et al., 2013, Habibi et al., 2013, Leitch et al., 2013) and reset human PSCs (Takashima et al., 2014). Immunostaining for 5-methylcytosine (5mC) is fainter in HNES cell nuclei compared with primed HNES cells (Figure S3A). Like reset PSCs, HNES cells show appreciable expression of TET1 and downregulation of de novo methyltransferase DNMT3B (Figures S3B and S3C). We performed whole-genome bisulfite sequencing on two HNES lines and their primed derivatives. Analysis confirmed genome-wide hypomethylation in male and female HNES cells, similar to levels of 25%–40% observed in human ICM and in contrast to >70% CpG methylation in conventional PSCs and primed HNES cells (Figure 2D). Both HNES lines showed extensive overlap in the distribution of CpG methylation sites (Figure 2E), with substantial hypomethylation compared with primed HNES cells (Figure 2F). The methylomes of HNES and reset H9 cells are very similar, suggesting that the epigenetic state of conventional human PSCs can be accurately and consistently reprogrammed. We analyzed methylation levels of CpG islands (CGIs) and performed PCA, revealing clustering of HNES with reset H9 cells and conventional human PSCs (H9) with primed HNES cells (Figure 2G). PC1 captured most of the variation (42%), indicating high resemblance between HNES cells and human ICMs (Guo et al., 2014). Comparisons of CGI methylation in HNES and H9 cells (Figure 2H) showed that the majority of CGIs are hypomethylated in both HNES cells and conventional PSCs, while many CGIs gain methylation in primed cells. Only a subset of CGIs is methylated in both conditions. These data highlight similarity between HNES and human ICM methylomes and show that conventional human PSCs have gained methylation at a number of CGIs when compared with HNES cells.

**Figure 3:**
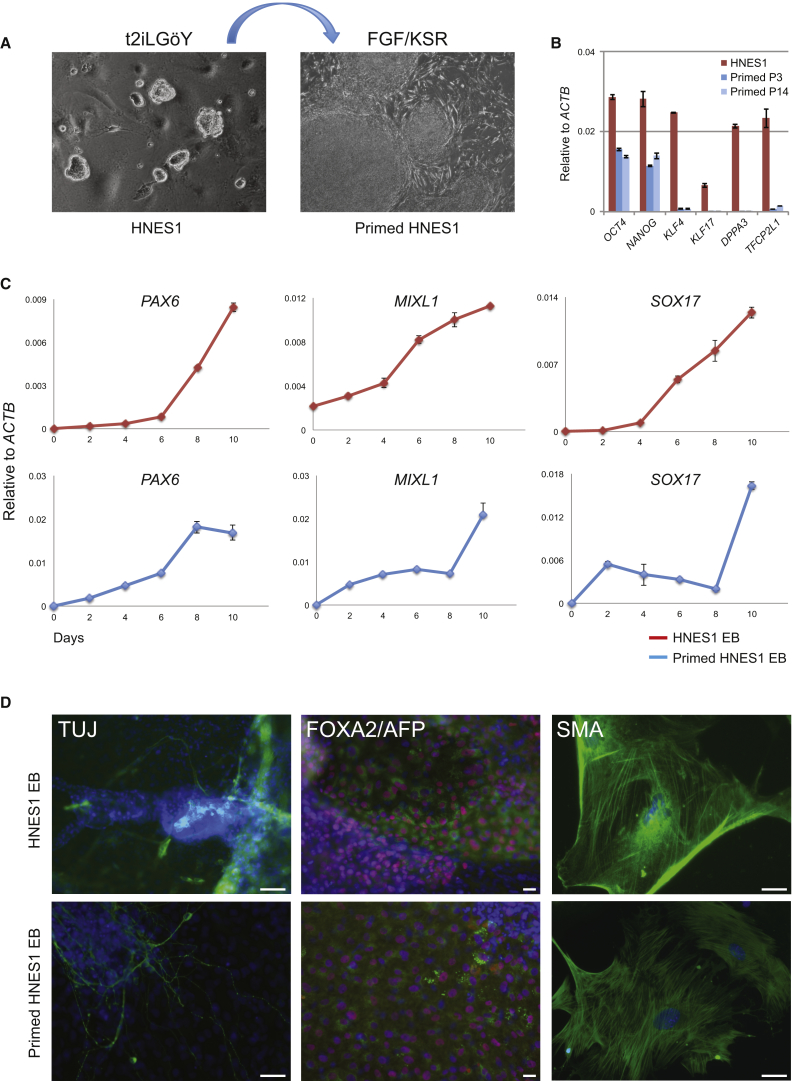
Differentiation (A) Colonies of naive HNES1 cells in t2iLGöY and primed HNES1 cells after 12 passages in FGF/KSR. (B) qRT-PCR analysis of naive marker expression in naive HNES1 cells and derivatives after three passages in FGF/KSR. Error bars indicate the SD of two independent reactions. (C) qRT-PCR analysis of embryoid bodies formed from HNES1 and primed HNES1 cells. Error bars indicate the SD two independent reactions. (D) Immunofluorescence of embryoid body outgrowths: TuJ1, β-III tubulin; AFP, α-fetoprotein; SMA, α-smooth muscle actin (green); FOXA2 (red). Nuclei (DAPI; blue). Scale bars, 100 μm.

We transferred HNES cells to conventional PSC culture medium containing FGF/KSR and lacking inhibitors. After one passage the domed colonies of HNES cells assumed flattened epithelial morphology, and after two passages resembled conventional PSC (Figure 3A). During this transition *OCT4* and *NANOG* were reduced, and naive markers, including *KLF17*, were extinguished (Figures 3B, S4A, and S4B).

We assessed whether HNES cells can undergo multilineage differentiation by generating embryoid bodies directly from naive and primed HNES cells. In both cases early lineage markers *PAX6*, *MIXL1*, and *SOX17* were upregulated (Figure 3C). Outgrowths from plated embryoid bodies displayed TuJ1-positive neuronal, FOXA2/AFP double-positive endoderm, and smooth muscle actin-positive cells (Figure 3D). We also applied a protocol for cardiomyocyte differentiation (van den Berg et al., 2016) to primed HNES cells and observed multiple regions of spontaneous contraction after 12 days (Movie S1). Cardiomyocyte identity was confirmed by expression of surface markers VCAM-1 and CD172a (SIRPα) (Figure S4C).

## Discussion

Hitherto, stem cell derivations from human embryos have yielded cells with features distinct from rodent ESCs and more similar to mouse post-implantation epiblast-derived stem cells (Brons et al., 2007, Tesar et al., 2007). This may be because the culture conditions used were inadequate to sustain naive pluripotency in the face of stimuli for developmental progression emanating from extraembryonic endoderm (Brook and Gardner, 1997) in ICM explants and/or provided by FGF and serum factors. Even for derivations commencing from single blastomeres, a blastocyst-like structure develops, followed by ICM outgrowth prior to cell line derivation (Taei et al., 2013). We show that after dissociation of the ICM to separate epiblast and primitive endoderm, stem cell colonies emerge directly in the presence of inhibitors of MAPK/Erk, GSK3, and PKC. Resulting HNES cell lines can be propagated by enzymatic dissociation to single cells, retain chromosomal integrity over many passages, exhibit features diagnostic of naive pluripotency, and are capable of multilineage differentiation.

Conventional human PSC cultures are heterogeneous, potentially comprising complex hierarchies (Davidson et al., 2015, Enver et al., 2009, Hough et al., 2014). Furthermore, pluripotency is an inherently plastic stage of development. It is unsurprising, therefore, that PSCs can adjust to alternative culture conditions with shifts in morphology and gene expression. Without objective criteria, these may be misinterpreted as a change in developmental status rather than accommodation to culture. In contrast, global transcriptome, metabolic properties, and DNA hypomethylation features align HNES cells with reset PSCs and distinguish them from conventional human PSCs. Of particular significance, HNES cells and reset PSCs express the naive pluripotency factors *KLF4*, *TFCP2L1*, *TBX3*, and *NANOG* found in the primate ICMs and functional in mouse ESC self-renewal. Additionally they express *KLF17*, which might compensate for lower expression of *KLF2*. Apart from NANOG, these factors are expressed at low levels or not at all in conventional human PSCs, including those variants purported to be naive by other criteria. We have shown that the reset PSC state is dependent on both KLF4 and TFCP2L1 (Takashima et al., 2014).

The naive gene regulatory network is not fully conserved between mouse and human. Absence of ESRRB marks a substantial distinction. Mouse ESCs can be maintained after deletion of *Esrrb* but are less stable (Martello et al., 2012). Lack of ESRRB may therefore render human naive PSC propagation inherently more demanding. Nonetheless, culture refinements and replacement of feeders with a defined substrate may be anticipated to facilitate their handling and possibly attainment of a ground state.

In summary, these findings suggest that it is possible to suspend human developmental progression at the pre-implantation epiblast phase and propagate a self-renewing pluripotent state analogous to mouse ESCs (Boroviak et al., 2014, Brook and Gardner, 1997). Derivation of equivalent cell lines from non-human primates and formation of high-contribution chimeras would provide further validation. However, our results support the case for naive pluripotency in human development and may reconcile the long-running debate about the difference between PSCs from mice and men.

## Experimental Procedures

### Embryo Manipulation

Supernumerary frozen human embryos were donated with informed consent under license from the UK HFEA. Embryos were thawed using EmbryoThaw medium (FertiPro) and cultured in drops of pre-equilibrated medium (Origio): EmbryoAssist for 1–8 cell stage (days 0–2), and BlastAssist for 8 cell stage to blastocyst (days 3–6) under embryo-tested mineral oil (Sigma). Expanded blastocysts (day 6) were subjected to immunosurgery (Pickering et al., 2005) to isolate ICMs using anti-human serum (Sigma). ICMs were treated with Accutase (Sigma or Gibco) for 5–10 min, and placed in a drop of medium for mechanical separation using a finely drawn Pasteur pipette. ICM cells were scattered onto mitotically inactivated (irradiated) murine embryonic fibroblasts (MEFs). Immunostaining was performed as described by Takashima et al. (2014).

### Naive Stem Cell Culture

Cells were propagated in modified N2B27 medium supplemented with PD0325901 (1 μM, prepared in-house), CHIR99021 (1 μM, prepared in-house), Gö6983 (2.5 μM, Sigma-Aldrich), rho-associated kinase inhibitor (Y-27632) (10 μM, Calbiochem), human LIF (10 ng/ml, prepared in-house), and ascorbic acid (250 μM, Sigma). N2B27 medium (1 l) comprised 490 ml of DMEM/F12 (Life Technologies), 490 ml of Neurobasal (Life Technologies), 10 ml of B27 (Life Technologies), 5 ml of N2 (prepared in-house), 10 μg/ml insulin (Sigma), 2 mM L-glutamine (Life Technologies), and 0.1 mM 2-mercaptoethanol (Sigma). N2 contains 100 μg/ml apo-transferrin (eBioscience, ABC2553), 3 μM sodium selenite (Sigma), 1.6 mg/ml putrescine (Sigma), and 2 μg/ml progesterone (Sigma) in DMEM/F12 (Life Technologies). Primary colonies and nascent cell lines were passaged manually as described above for ICMs. Established cells were passaged either manually with Accutase (Life Technologies) dissociation reagent or as a pool using TrypLE Express (Life Technologies). Cells were cultured in 5% O_2_ and 7% CO_2_ in a humidified incubator at 37°C. Cells were frozen in 50% t2iLGöY medium with 40% serum and 10% DMSO.

### Conversion to Primed Pluripotency

HNES cells were seeded on MEFs in t2iLGöY for 24 hr, then transferred into FGF/KSR medium for 7–10 days before passaging with TrypLE Express. Y-27632 was added for the first passage. Thereafter cells were passaged as clusters using collagenase/dispase (Roche). FGF/KSR medium comprised 20% KnockOut Serum Replacement (Invitrogen), 1× non-essential amino acids (Invitrogen), 2 mM L-glutamine (Invitrogen), 100 μM 2-mercaptoethanol (Sigma), 10 ng/ml FGF2 (prepared in-house), and DMEM/F-12 basal medium (Sigma-Aldrich). Established primed HNES cultures can also be maintained in mTeSR1 or E8 media (StemCell Technologies) on Matrigel.

### In Vitro Differentiation

HNES cells or primed derivatives were dissociated with TrypLE Express and placed in PrimeSurface 96V cell plates (Sumitomo Bakelite MS-9096V) at a density of 4,000–5,000 cells per well in medium containing 20% KSR. Y-27632 was added during the first 24 hr of aggregation. At day 7 aggregates were plated on gelatin in 20% FBS.

Cardiomyocyte differentiation was performed as described by van den Berg et al. (2016).

## Author Contributions

J.N., A.S., and G.G. planned the study; G.G., F.v.M., F.S., Y.C., P.B., and J.N. carried out experiments and analyses; P.B. performed RNA sequencing and bioinformatics; W.R. supervised methylome studies; J.N., A.S., P.B., and G.G. prepared the manuscript in consultation with all authors.

## Figures and Tables

**Table 1:** Derivation of Naive Epiblast Stem Cell Lines

Experiment	Embryos Surviving Thaw	Blastocysts^a^	Dissociated ICMs	Cell Lines	Cumulative Passages
1	24	4	1	HNES1	P30
2	9	4	2	HNES2	P22
HNES3	P29
3	20	4	4	HNES4^b^	P21
4	5	2	1	c	
Total	58	14	8	4	

a Embryos cavitated by day 6.

b Primary colonies lost in three cases associated with incubator humidity failure.

c Primary colonies emerged but failed to expand after five passages.

## Accession Numbers

Data from this study are available in ArrayExpress: E-MTAB-4461 (RNA-seq), E-MTAB-4462 (BS-seq) and E-MTAB-4463 (Affymetrix).

## Supplemental Information

Document S1. Supplemental Experimental Procedures, Figures S1–S4, and Tables S1–S3

Table S4. RNA-Seq Read Counts for All Samples, Related to Figures 2 and S3

Table S5. FPKM Values for Conventional RNA-Seq Data, Related to Figures 2 and S3

Table S6. FPKM Values for Single-Cell RNA-Seq Data, Related to Figures 2 and S3

Table S7. FPKM Values for All Samples Analyzed, Related to Figures 2 and S3

Movie S1. Cardiomyocyte Differentiation

Document S2. Article plus Supplemental Information
