# Supplementary material for: Naive Pluripotent Stem Cells Derived Directly from Isolated Cells of the Human Inner Cell Mass
Source: Stem Cell Reports. 2016 Mar 3;6(4):437–46. doi: 10.1016/j.stemcr.2016.02.005 (PMC4834040; doi:10.1016/j.stemcr.2016.02.005)
Supplement: Document S1. Supplemental Experimental Procedures, Figures S1–S4, and Tables S1–S3 [file mmc1.pdf]

**Stem Cell Reports, Volume *6***

## Supplemental Information

# Naive Pluripotent Stem Cells Derived Directly from Isolated Cells of the Human Inner Cell Mass

**Ge Guo, Ferdinand von Meyenn, Fatima Santos, Yaoyao Chen, Wolf Reik, Paul Bertone, Austin Smith, and Jennifer Nichols**

## Supplemental Figures

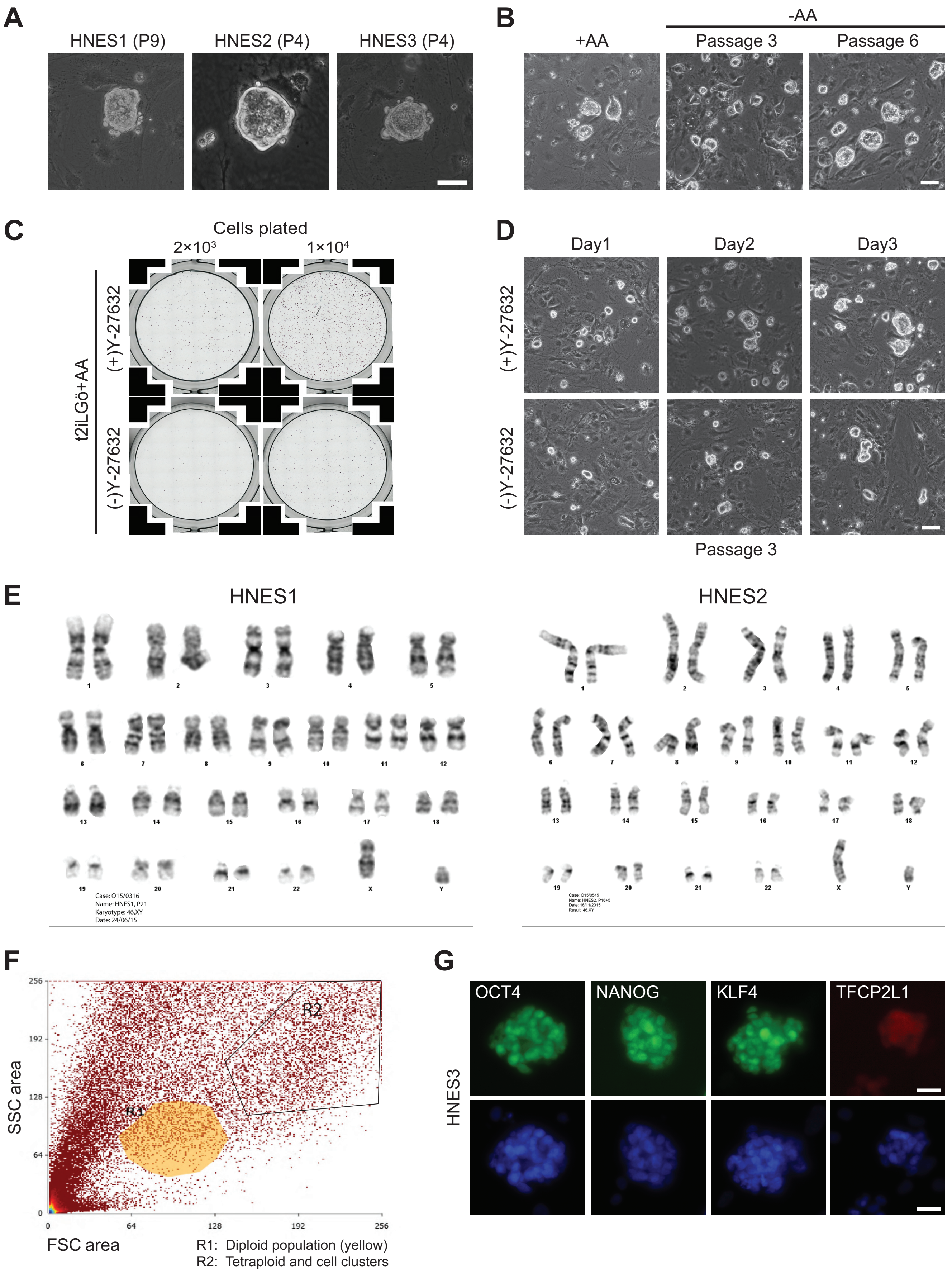


**Figure S1, related to Figure 1:** (A) Colonies of HNES cells. Passage numbers are shown in brackets. (B) HNES1 cells in t2iLGö with or without ascorbic acid (AA). (C) HNES1 colony formation assay in t2iLGöY or after withdrawal of ROCK inhibitor (Y-27632). (D) HNES1 cells expanding after three passages with or without ROCK inhibitor. (E) Karyotype analysis of HNES1 and HNES2 cells by Giemsa staining. (F) Density plot showing HNES2 diploid cells (R1) sorted by flow cytometry. (G) Immunofluorescence staining of pluripotency markers. Scale bar, 25 µm.

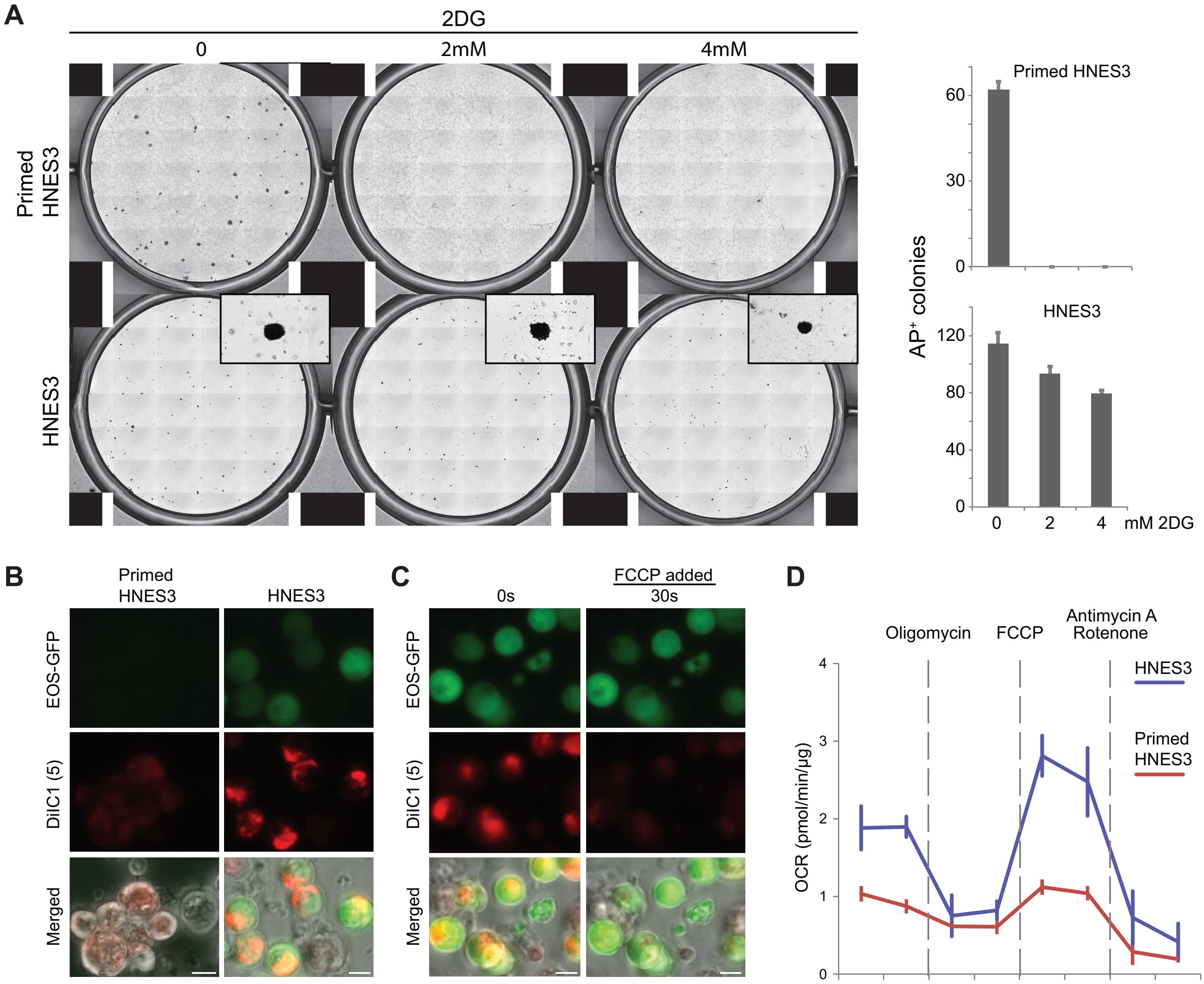


**Figure S2, related to Results:** (A) Colony formation assay in culture conditions supplemented with the indicated concentrations of 2-deoxy-D-glucose (2DG). Quantification, right. Error bars indicate standard deviation (s.d.) of two replicates. (B) Mitochondrial activity staining using mitochondrial membrane potential-dependent MitoProbe DiIC1 (5). (C) Stain intensity decreases after cells are treated with the mitochondrial uncoupling reagent carbonyl cyanide-4-(trifluoromethoxy)phenylhydrazone (FCCP), confirming signal specificity. (D) Oxygen consumption rate (OCR) measured by the XF Cell Mito Stress Test assay provides a complete mitochondrial profile (see Extended Experimental Procedures). HNES cells exhibit higher OCR relative to primed PSC, particularly at elevated mitochondrial respiration following exposure to FCCP. Error bars indicate s.d. of five replicates.

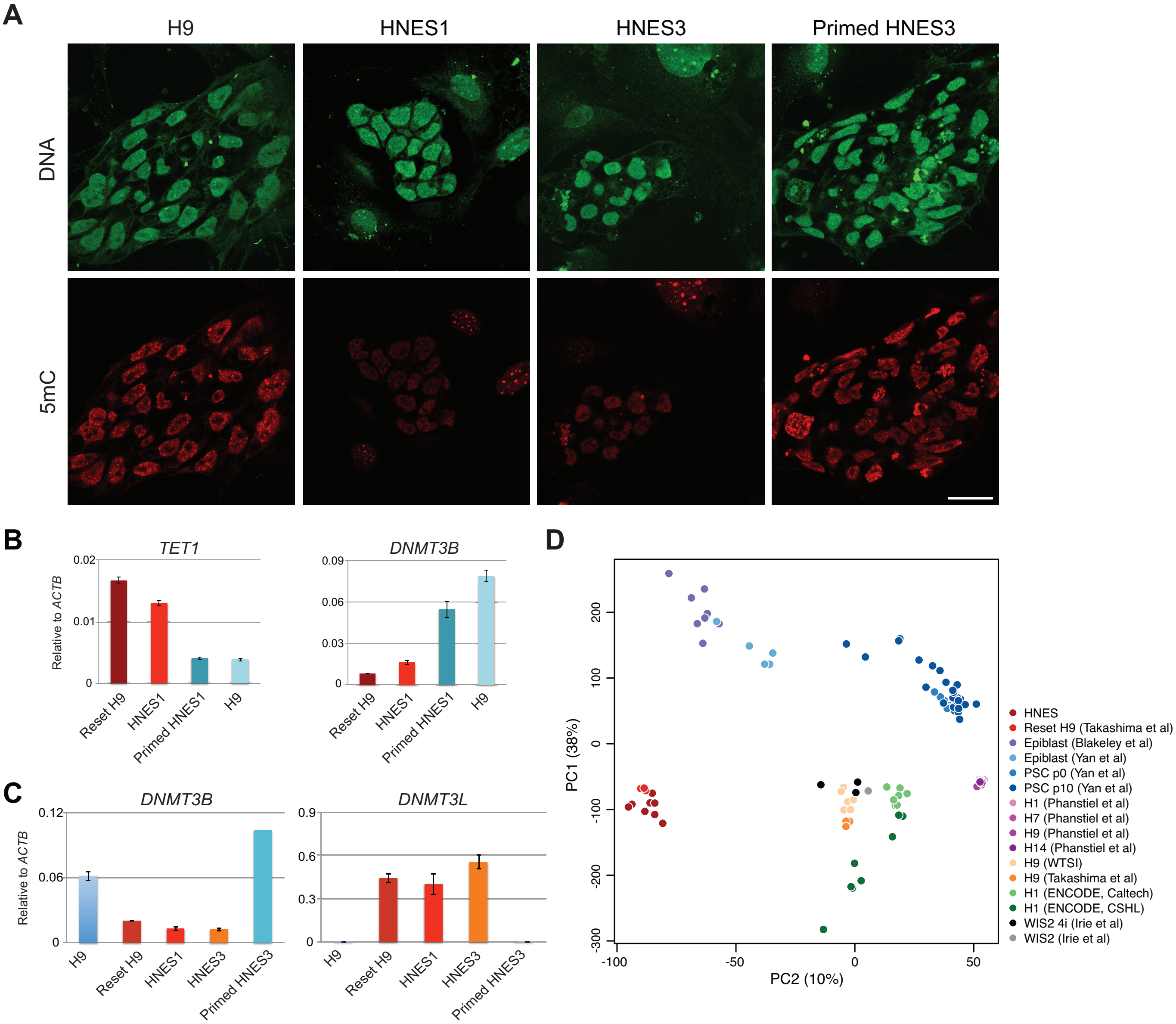


**Figure S3, related to Figure 2:** (A) Immunofluorescence staining of 5mC (red). DNA was counterstained with YOYO-1 (green). Scale bar, 20 µm. (B, C) qRT-PCR analysis of human pluripotent cell lines for TET1 and DNA methyltransferases. Error bars indicate s.d. of two independent reactions. (D) Principal component analysis of samples represented in Figure 2B including WIS2 human PSC cultured in conventional and modified NHSM (4i) conditions (Irie et al., 2015).

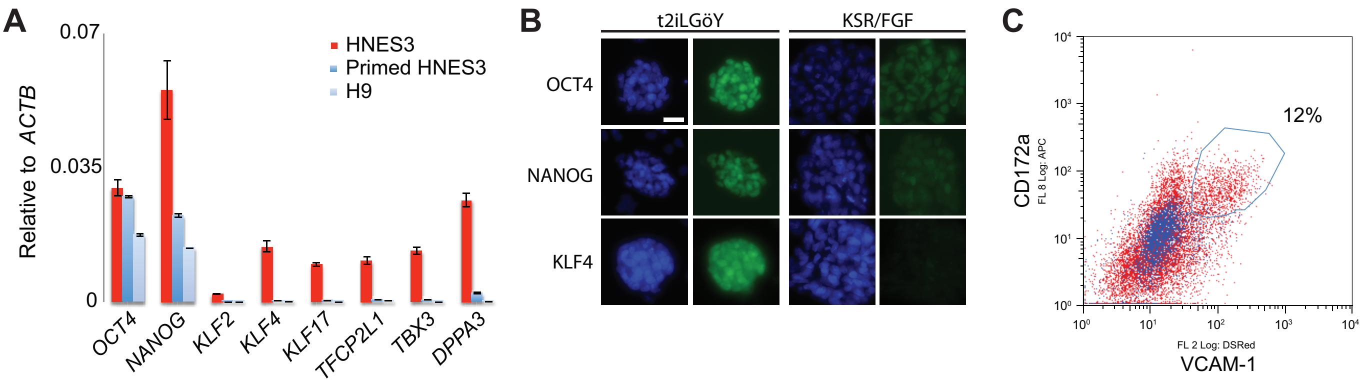


**Figure S4, related to Figure 3:** (A) qRT-PCR analysis showing reduced expression of naïve pluripotency markers in primed HNES3 and H9 cultures compared to naïve HNES3 cells. Error bars indicate s.d. of two independent reactions. (B) Immunofluorescence staining showing loss of expression of KLF4 in primed HNES3 cells. Scale bar, 25µm. (C) Flow cytometry analysis to isolate the CD172a and VCAM-1 double-positive cardiomyocyte population following differentiation for 12 days.

## Supplemental Tables

**Table S1, related to Table 1:** Chromosomal analysis of HNES cell lines.

| Cell line | Passage | Karyotype |
|---|---|---|
| HNES1* | P11<br>P21 | 46 XY (20/20)<br>46 XY (11/11) |
| HNES2 | P6<br>P16+5 | 46 XY (10/30); tetraploid-like (20/30)<br>46 XY (20/20) |
| HNES3 | P18 | 46 XX (6/20); 47 XX (14/20),+22^ |
| HNES4 | P20 | 48 XY, +i(12)(P10),+i(12)(P10) |

* HNES1 cells were also examined by array CGH after 14 passages cultured in t2iLGöY and a further 8 passages in FGF/KSR. This analysis confirmed a 46 XY chromosome complement with no detectable abnormalities.

^ Heterogeneity for trisomy 22 observed in HNES3 may reflect mosaicism in the original ICM. Trisomy 22 is among the most frequently occurring chromosomal abnormalities causing spontaneous abortion during the first trimester (Nagaishi et al., 2004; Sasabe et al., 1999).

**Table S2, related to Figures 1, 3 and S4:** TaqMan qPCR assays.

| Gene | TaqMan assay |
|---|---|
| ACTB | Hs01060665_g1 |
| POU5F1 | Hs01654807_s1 |
| NANOG | Hs02387400_g1 |
| KLF17 | Hs00703004_s1 |
| KLF4 | Hs00358836_m1 |
| KLF2 | Hs00360439_g1 |
| DPPA3 | Hs01931905_g1 |
| TFCP2L1 | Hs00232708_m1 |
| TBX3 | Hs00195612_m1 |
| DNMT3B | Hs00171876_m1 |
| DNMT3L | Hs01081364_m1 |
| TET1 | Hs00286756_m1 |
| FOXA2 | Hs00232764_m1 |
| PAX6 | Hs00240871_m1 |
| SOX17 | Hs00751752_s1 |
| MIXL1 | Hs00430824_g1 |

**Table S3, related to Figures 1, 3, S1, S3 and S4:** Primary antibodies used in this study.

| Antibody | Vendor | Number | Dilution |
|---|---|---|---|
| OCT3/4 | Santa Cruz | sc-5279 | 1:200 |
| NANOG | eBioscience | 14-5769-82 | 1:200 |
| KLF4 | Santa Cruz | sc-20691 | 1:400 |
| TFCP2L1 | R&D | AF5726 | 1:500 |
| TUJ1 | R&D | MAB1195 | 1:200 |
| KLF17 | ATLAS Antibodies | HPA024629 | 1:500 |
| 5-mC | Eurogentec | BI-MECY-0100 | 1:250 |
| AFP | Abcam | ab169552 | 1:100 |
| FOXA2 | R&D | AF2400 | 1:100 |
| SMA | Sigma | A2547 | 1:200 |
| APC-CD172a | Miltenyi Biotec | 130-099-785 | |
| PE-anti-human CD106 (VCAM-1) | BioLegend | 305806 | |

**Tables S4–S7, related to Figures 2 and S3:** RNA-seq expression data (available online).

## Supplemental Experimental Procedures

### Colony formation assays

1000 HNES or conventional PSCs were seeded in each well of a 12-well plate. Cultures were fixed and stained for alkaline phosphatase at day 7. Images were acquired automatically with the ProScan II system (Prior Scientific) and quantified with the cell counter from ImageJ. For ROCK inhibitor (Y-27632) withdrawal assays, $2\times10^3$ or $1\times10^4$ cells were seeded in each well of a 12-well plate in t2iLGö with or without Y-27632 for five days. Cultures were stained for alkaline phosphatase and images acquired with ProScan II.

### Cardiomyocyte differentiation

Primed HNES cells were explanted into 6-well plates at approximately 30% confluence. Two to three days later (~70% confluence) differentiation medium was administered following the protocol described previously (van den Berg et al., 2016). APC-CD172a (Miltenyi Biotec, 130-099-785) and PE-anti-human CD106 (VCAM-1) (BioLegend, 130-099-785) were used to identify cardiomyocytes by flow cytometry.

### Embryoid body (EB) differentiation

Differentiation medium comprised GMEM (Sigma G5154) with L-glutamine (2 mM), sodium pyruvate (1 mM), 2-mercaptoethanol (0.1 mM), non-essential amino acids (1×, Life Technologies) plus 20% fetal bovine serum for primed PSC or 20% KnockOut Serum Replacement (KSR) for HNES cells. 10 µM ROCK inhibitor was added for the first day of aggregation. Cultures were maintained in 5% $O_2$ and 7% $CO_2$ in a humidified incubator at 37°C. QIAshredder (QIAGEN) was used to homogenize EB samples for RNA extraction.

### Immunofluorescence staining

Immunostaining on cultured cells and EB outgrowths were performed using standard protocols. Briefly, samples were fixed in 4% PFA and permeabilized with PBS/0.3% Triton-X100. Following one hour of blocking in PBS/0.1% Triton-X100/5% donkey serum, primary antibody staining was carried out in the blocking solution. 5mC staining was performed as described (Ficz et al., 2013). Antibodies used in this study were: OCT3/4 (Santa Cruz sc-5279 (1:200 dilution)), NANOG (eBioscience 14-5769-82 (1:200)), KLF4 (Santa Cruz sc-20691 (1:400)), TFCP2L1 (R&D AF5726 (1:500)), KLF17 (ATLAS Antibodies HPA024629 (1:500)), AFP (Abcam ab169552 (1:100)), FOXA2 (R&D AF2400 (1:100)), SMA (Sigma A2547 (1:200)), 5-mC (Eurogentec BI-MECY-0100 (1:250)).

### Karyotype analysis

G-banding karyotype and CGH array analyses were performed at Medical Genetics Laboratories, Cambridge University Hospitals NHS Foundation Trust following their standard protocols. Genomic DNA was isolated with the Qiagen Blood & Tissue kit and profiled on the Affymetrix CytoScan 750K SNP genotyping array. Chromosomal integrity was assessed with the Affymetrix Chromosome Analysis Suite (ChAS) based on human genome build GRCh37/hg19. Data are available in the ArrayExpress repository under accession E-MTAB-4463.

### Flow cytometry

Flow cytometry analyses were performed with a Dako Cytomation CyAn ADP high-performance cytometer with Summit software. Data were analyzed using FlowJo.

### Reverse transcription and real-time PCR

Total RNA was extracted with the RNAeasy kit (QIAGEN). cDNA was synthesized using SuperScript III (Invitrogen) with oligo-dT adapter primers (GACCACGCGTATCGATGTCGACTTTTTTTTTTTTTTTT). PCR was performed with the TaqMan assays listed in Supplementary Table 2.

**Transcriptome sequencing**

Feeder cells were depleted by culture on gelatin-coated dishes for 40 minutes and harvesting cells in suspension. Total RNA was extracted with the TRIzol/chloroform method (Invitrogen), followed by re-suspension in RNAsecure (Ambion), incubation with TURBO DNase (Ambion) at 37ºC for 1 hour, and further phenol/chloroform extraction and ethanol precipitation. RNA integrity was assessed with the RNA 6000 Nano assay on the 2100 Bioanalyzer (Agilent). Ribosomal RNA was depleted from 5 µg of total RNA using Ribo-Zero capture probes (Epicentre). RNA samples were sheared by ultrasonication on a Covaris S2 for 90 s set at Duty Cycle 10, Cycles per Burst 200 and Intensity 5. Fragmented RNA was reverse-transcribed with a combination of random hexamer and oligo-dT primers (New England Biolabs) by SuperScript III (Invitrogen) at 50ºC for 2 hours in the presence of 6 µg/mL actinomycin D (Sigma) to inhibit formation of second-strand products (Perocchi et al., 2007). Second-strand cDNA was synthesized by DNA Polymerase I in the presence of RNase H with dUTPs substituted for dTTPs at 16ºC for 2 hours. Sequential end repair and 30′-adenylation of cDNA products were carried out with T4 DNA polymerase and T4 polynucleotide kinase (20ºC), and with exo$^-$ Klenow fragment (65ºC) in the presence of dATPs (New England BioLabs). Products were ligated to barcoded adapters (NEXTflex-96, Bioo Scientific) by T4 DNA ligase (New England BioLabs) at 20ºC for 30 min. Second-strand DNA was digested with uracil DNA glycosylase (UDG) and Endonuclease VIII at 37ºC for 30 min. PCR amplification of first-strand library constructs was carried out with KAPA HiFi DNA polymerase (KAPA Biosystems) for 13 cycles using denaturing and annealing conditions optimized for even A/T versus G/C processing (Aird et al., 2011). Purification of reaction products at each step was performed with Ampure XP paramagnetic beads (Beckman Coulter). Library size distribution and molarity were assessed by DNA 1000 micrifluidic chips on the 2100 Bioanalyzer (Agilent). Sequencing was performed on the Illumina HiSeq 2500 in 125bp paired-end format. Data are available from ArrayExpress and ENA via accession E-MTAB-4461.

**RNA-seq data analysis**

Additional RNA-seq data from published studies were obtained from the European Nucleotide Archive (ENA (Silvester et al., 2015)). Data were compiled from conventional and reset H9 cells previously described (accession ERP006823, (Takashima et al., 2014)), H1 cells sequenced by the ENCODE Consortium (SRP014320, (Djebali et al., 2012)), H9 cells forming part of a comparative study of pluripotent cells at the Wellcome Trust Sanger Institute (ERP007180), H1, H7, H9 and H14 cells cultured in the Thomson lab (http://scor.chem.wisc.edu (Phanstiel et al., 2011)), WIS2 cells in conventional and modified NHSM/4i conditions (SRP045294, (Irie et al., 2015)) and early human embryo cells and explant PSC cultures profiled by single-cell RNA-seq (SRP011546, (Yan et al., 2013) and SRP055810, (Blakeley et al., 2015)). Residual adapter sequences were removed with Cutadapt (Martin, 2011) and reads were aligned to human genome build GRCh38/hg38 with STAR 2.4.2a (Dobin et al., 2013) using the two-pass method for novel splice detection (Engström et al., 2013). Read alignment was guided by GENCODE v23 (Harrow et al., 2012) human gene annotation from Ensembl release 82 (Cunningham et al., 2015) and splice junction donor/acceptor overlap settings were tailored to the corresponding read length of each dataset. Transcripts were quantified with htseq-count (Anders et al., 2015) based on annotation from Ensembl 82. Duplicate entries (1893 of 60619, primarily Y and snoRNAs) were consolidated to retain the transcript with maximum read coverage as the representative gene product. Libraries were corrected for total read counts using size factors computed by the Bioconductor package DESeq (Anders and Huber, 2010). Principal components were computed by singular value decomposition with the prcomp function in the R stats package from variance-stabilized count data. For PCA with asynchronous single-cell data, cell cycle-associated genes in GO:0007049 (208) were omitted, as were genes that registered zero counts in all single-cell samples compared (24611 of 58726 unique). The remaining 25000 most variable genes were used for PCA. For display of expression data, counts were normalized for gene length to yield FPKM values and scaled to the mean expression over all samples. Heatmaps include genes for which a difference in expression was observed (i.e., scaled expression > 1 or < -1 in at least one sample).

**Bisulfite sequencing**

Post-bisulfite adapter tagging (PBAT) libraries were prepared as previously described (Smallwood et al., 2014) with some modifications. Feeder cells were depleted by culture on gelatin-coated dishes for 40 minutes and harvesting cells in suspension. Pelleted cells were treated with lysis buffer (10 mM Tris-Cl pH 7.4 and 2% SDS) and 0.5 µl proteinase K followed by incubation at 37°C for 1 hour. Bisulfite conversion was performed on cell lysates using the EZ DNA Methylation-Direct MagPrep Kit (Zymo). After purification, first-strand synthesis was performed using 6N-forward oligos for 37°C for 30 min. Subsequently, samples were treated with Exonuclease I at 37°C for 1 hour, before DNA was purified using 0.8× Agencourt Ampure XP beads (Beckman Coulter). Samples were eluted in second-strand synthesis mix with 6N-reverse oligo and incubated at 37°C for 90 min. DNA was purified using 0.8× Agencourt Ampure XP beads and amplified with KAPA HiFi HotStart DNA Polymerase (KAPA Biosystems) using indexed iPCRTag primers (Quail et al., 2012). Amplified libraries were pooled, purified using 0.8× Agencourt Ampure XP beads, and assessed for quality and quantity using High-Sensitivity DNA chips on the 2100 Bioanalyzer (Agilent) and the KAPA Library Quantification Kit for Illumina (KAPA Biosystems). Libraries were sequenced in 150bp single-end format on the Illumina MiSeq or 125bp paired-end on the Illumina HiSeq 2500. Data are available from ArrayExpress and ENA via accession E-MTAB-4462.

**BS-seq data analysis**

Sequencing reads were processed to remove the first 6 bases, adapter sequences and poor-quality reads using Trim Galore v0.3.8 (http://www.bioinformatics.babraham.ac.uk/projects/trim_galore, parameters: --clip_r1 6). The remaining sequences were mapped to human genome build GRCh37/hg19 using Bismark v0.13.1 (parameters: --bowtie2 --pbat) (Krueger and Andrews, 2011), and CpG methylation calls were extracted and analysed using SeqMonk (www.bioinformatics.babraham.ac.uk/projects/seqmonk) and custom R scripts. Global comparison of methylation between samples was calculated by averaging CpG methylation levels over 500kb windows.

**Oxygen consumption rate (OCR)**

OCR was measured using an XF24 Analyzer (Seahorse Bioscience). Seahorse plates were pre-treated with Cell-Tak Cell and Tissue Adhesive (Corning, 4 µg/cm$^2$) according to the manufacturer's protocol, and 200,000 cells were seeded on each well immediately prior to the experiment. Culture medium was replaced by XF Base Medium (Seahorse Bioscience) supplemented with 2 mM sodium pyruvate and 10 mM glucose with an adjusted pH of 7.4. Cells were then incubated at 37°C in atmospheric $CO_2$ for one hour. Four compounds from the XF Cell Mito Stress Test kit (Seahorse Bioscience) were injected during the assay to achieve the following final concentrations: oligomycin (1 mM), FCCP (0.5mM), antimycin A (1 mM) and rotenone (1 mM). $O_2$ consumption was subsequently normalized to the protein content of each well.

**Mitochondrial membrane potential**

Dissociated cells were plated in growth medium onto Ibidi µ-plates pre-treated with Cell-Tak adhesive reagent (Roche). MitoProbe DiIC1 (5) (Life Technologies) was added at 50 mM and incubated at growth condition for 30 min before imaging (Leica DMI3000). After initial imaging, the uncoupling agent FCCP (0.5 mM) was added and samples were imaged again to determine background staining.

## References

Aird, D., Ross, M.G., Chen, W.S., Danielsson, M., Fennell, T., Russ, C., Jaffe, D.B., Nusbaum, C., and Gnirke, A. (2011). Analyzing and minimizing PCR amplification bias in Illumina sequencing libraries. Genome Biol *12*, R18.

Anders, S., and Huber, W. (2010). Differential expression analysis for sequence count data. Genome Biol *11*, R106.

Anders, S., Pyl, P.T., and Huber, W. (2015). HTSeq--a Python framework to work with high-throughput sequencing data. Bioinformatics *31*, 166-169.

Blakeley, P., Fogarty, N.M., Del Valle, I., Wamaitha, S.E., Hu, T.X., Elder, K., Snell, P., Christie, L., Robson, P., and Niakan, K.K. (2015). Defining the three cell lineages of the human blastocyst by single-cell RNA-seq. Development *142*, 3151-3165.

Cunningham, F., Amode, M.R., Barrell, D., Beal, K., Billis, K., Brent, S., Carvalho-Silva, D., Clapham, P., Coates, G., Fitzgerald, S.*, et al.* (2015). Ensembl 2015. Nucleic Acids Res *43*, D662-669.

Djebali, S., Davis, C.A., Merkel, A., Dobin, A., Lassmann, T., Mortazavi, A., Tanzer, A., Lagarde, J., Lin, W., Schlesinger, F.*, et al.* (2012). Landscape of transcription in human cells. Nature *489*, 101-108.

Dobin, A., Davis, C.A., Schlesinger, F., Drenkow, J., Zaleski, C., Jha, S., Batut, P., Chaisson, M., and Gingeras, T.R. (2013). STAR: ultrafast universal RNA-seq aligner. Bioinformatics *29*, 15-21.

Engström, P.G., Steijger, T., Sipos, B., Grant, G.R., Kahles, A., Ratsch, G., Goldman, N., Hubbard, T.J., Harrow, J., Guigo, R.*, et al.* (2013). Systematic evaluation of spliced alignment programs for RNA-seq data. Nat Methods *10*, 1185-1191.

Ficz, G., Hore, T.A., Santos, F., Lee, H.J., Dean, W., Arand, J., Krueger, F., Oxley, D., Paul, Y.L., Walter, J.*, et al.* (2013). FGF Signaling Inhibition in ESCs Drives Rapid Genome-wide Demethylation to the Epigenetic Ground State of Pluripotency. Cell Stem Cell *13*, 351-359.

Harrow, J., Frankish, A., Gonzalez, J.M., Tapanari, E., Diekhans, M., Kokocinski, F., Aken, B.L., Barrell, D., Zadissa, A., Searle, S.*, et al.* (2012). GENCODE: the reference human genome annotation for The ENCODE Project. Genome Res *22*, 1760-1774.

Irie, N., Weinberger, L., Tang, W.W., Kobayashi, T., Viukov, S., Manor, Y.S., Dietmann, S., Hanna, J.H., and Surani, M.A. (2015). SOX17 is a critical specifier of human primordial germ cell fate. Cell *160*, 253-268.

Krueger, F., and Andrews, S.R. (2011). Bismark: a flexible aligner and methylation caller for Bisulfite-Seq applications. Bioinformatics *27*, 1571-1572.

Martin, M. (2011). Cutadapt removes adapter sequences from high-throughput sequencing reads. EMBnet *17*, 10-12.

Perocchi, F., Xu, Z., Clauder-Munster, S., and Steinmetz, L.M. (2007). Antisense artifacts in transcriptome microarray experiments are resolved by actinomycin D. Nucleic Acids Res *35*, e128.

Phanstiel, D.H., Brumbaugh, J., Wenger, C.D., Tian, S., Probasco, M.D., Bailey, D.J., Swaney, D.L., Tervo, M.A., Bolin, J.M., Ruotti, V.*, et al.* (2011). Proteomic and phosphoproteomic comparison of human ES and iPS cells. Nat Methods *8*, 821-827.

Quail, M.A., Otto, T.D., Gu, Y., Harris, S.R., Skelly, T.F., McQuillan, J.A., Swerdlow, H.P., and Oyola, S.O. (2012). Optimal enzymes for amplifying sequencing libraries. Nat Methods *9*, 10-11.

Silvester, N., Alako, B., Amid, C., Cerdeno-Tarraga, A., Cleland, I., Gibson, R., Goodgame, N., Ten Hoopen, P., Kay, S., Leinonen, R.*, et al.* (2015). Content discovery and retrieval services at the European Nucleotide Archive. Nucleic Acids Res *43*, D23-29.

Smallwood, S.A., Lee, H.J., Angermueller, C., Krueger, F., Saadeh, H., Peat, J., Andrews, S.R., Stegle, O., Reik, W., and Kelsey, G. (2014). Single-cell genome-wide bisulfite sequencing for assessing epigenetic heterogeneity. Nat Methods *11*, 817-820.

Takashima, Y., Guo, G., Loos, R., Nichols, J., Ficz, G., Krueger, F., Oxley, D., Santos, F., Clarke, J., Mansfield, W.*, et al.* (2014). Resetting transcription factor control circuitry toward ground-state pluripotency in human. Cell *158*, 1254-1269.

van den Berg, C.W., Elliott, D.A., Braam, S.R., Mummery, C.L., and Davis, R.P. (2016). Differentiation of Human Pluripotent Stem Cells to Cardiomyocytes Under Defined Conditions. Methods Mol Biol *1353*, 163-180.

Yan, L., Yang, M., Guo, H., Yang, L., Wu, J., Li, R., Liu, P., Lian, Y., Zheng, X., Yan, J.*, et al.* (2013). Single-cell RNA-Seq profiling of human preimplantation embryos and embryonic stem cells. Nat Struct Mol Biol *20*, 1131-1139.
