# Supplementary figures and images for: Naive Pluripotent Stem Cells Derived Directly from Isolated Cells of the Human Inner Cell Mass
Source: Stem Cell Reports. 2016 Mar 3;6(4):437–46. doi: 10.1016/j.stemcr.2016.02.005 (PMC4834040; doi:10.1016/j.stemcr.2016.02.005)

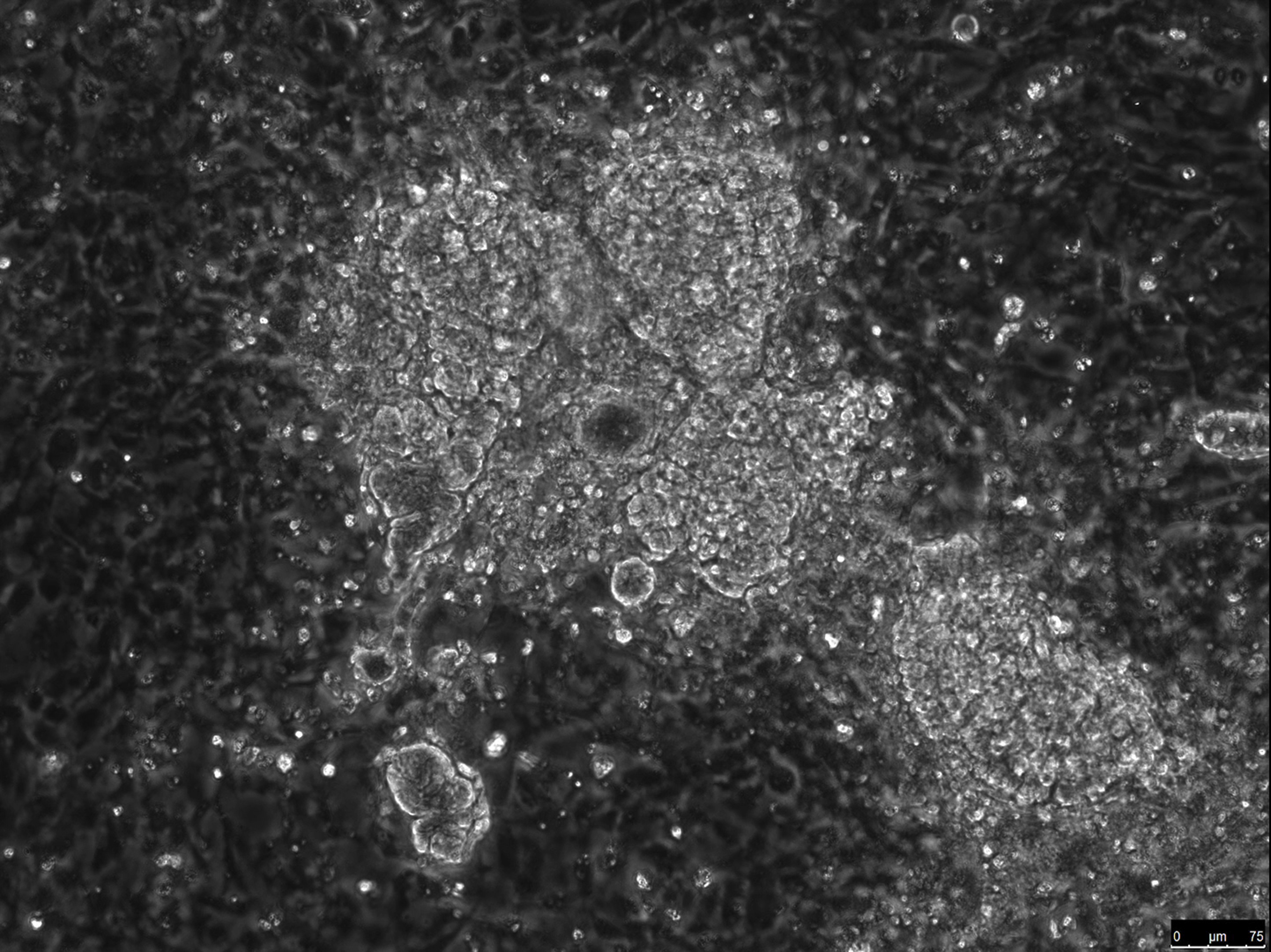

Supplement: Movie S1. Cardiomyocyte Differentiation [file mmc6.jpg]
