# Supplementary material for: Naive Pluripotent Stem Cells Derived Directly from Isolated Cells of the Human Inner Cell Mass
Source: Stem Cell Reports. 2016 Mar 3;6(4):437–46. doi: 10.1016/j.stemcr.2016.02.005 (PMC4834040; doi:10.1016/j.stemcr.2016.02.005)
Supplement: Document S2. Article plus Supplemental Information [file mmc7.pdf]

Stem Cell Reports
Report
OPEN ACCESS

Naive Pluripotent Stem Cells Derived Directly from Isolated Cells of the Human Inner Cell Mass

Ge Guo,[1] Ferdinand von Meyenn,[4] Fatima Santos,[4] Yaoyao Chen,[1] Wolf Reik,[4,5] Paul Bertone,[1] Austin Smith,[1,3,*] and Jennifer Nichols[1,2,*]
[1]Wellcome Trust – Medical Research Council Stem Cell Institute, University of Cambridge, Tennis Court Road, Cambridge CB2 1QR, UK
[2]Department of Physiology, Development and Neuroscience, University of Cambridge, Downing Street, Cambridge CB2 4BG, UK
[3]Department of Biochemistry, University of Cambridge, Tennis Court Road, Cambridge CB2 1GA, UK
[4]Epigenetics Programme, Babraham Institute, Cambridge CB22 3AT, UK
[5]Wellcome Trust Sanger Institute, Hinxton, Cambridge CB10 1SA, UK
*Correspondence: austin.smith@cscr.cam.ac.uk (A.S.), jn270@cam.ac.uk (J.N.)
http://dx.doi.org/10.1016/j.stemcr.2016.02.005
This is an open access article under the CC BY license (http://creativecommons.org/licenses/by/4.0/).

SUMMARY

Conventional generation of stem cells from human blastocysts produces a developmentally advanced, or primed, stage of pluripotency. In vitro resetting to a more naive phenotype has been reported. However, whether the reset culture conditions of selective kinase inhibition can enable capture of naive epiblast cells directly from the embryo has not been determined. Here, we show that in these specific conditions individual inner cell mass cells grow into colonies that may then be expanded over multiple passages while retaining a diploid karyotype and naive properties. The cells express hallmark naive pluripotency factors and additionally display features of mitochondrial respiration, global gene expression, and genome-wide hypomethylation distinct from primed cells. They transition through primed pluripotency into somatic lineage differentiation. Collectively these attributes suggest classification as human naive embryonic stem cells. Human counterparts of canonical mouse embryonic stem cells would argue for conservation in the phased progression of pluripotency in mammals.

INTRODUCTION

Human pluripotent stem cells (PSCs), whether derived from blastocysts or generated by somatic cell reprogramming, differ substantially from canonical mouse embryonic stem cells (ESCs) and are considered to represent a later phase of epiblast development, termed primed pluripotency (Hackett and Surani, 2014; Nichols and Smith, 2009; Rossant, 2015). Multiple claims of conversion of primed human PSCs into a more naive-like phenotype have been published (reviewed in (Davidson et al., 2015)). These reports are based on a shift in some attribute(s) in response to exogenous reprogramming factors and/or altered culture conditions. Evidence has been lacking, however, for a global state that correlates with mouse ESCs or human naive epiblast (Huang et al., 2014), or for presence of a functional gene regulatory network to sustain naive pluripotency (Boroviak et al., 2015; Dunn et al., 2014; Martello and Smith, 2014).

Two independent studies have described resetting of human PSCs to resemble mouse ESCs following short-term expression of *KLF2* and *NANOG* (Takashima et al., 2014; Theunissen et al., 2014). Reset cells are maintained in medium based on components used for mouse ESCs (Dutta et al., 2011; Ying et al., 2008) comprising titrated inhibition of glycogen synthase kinase-3 and blockade of the mitogen-activated protein kinase (MAPK/Erk) pathway (t2i) with leukemia inhibitory factor (LIF), plus protein kinase C (PKC) inhibition (Takashima et al., 2014). LIF and t2i have also been used to achieve resetting in combination with activin plus inhibitors of BRaf, Src family kinases, and Rho-associated kinase (ROCK) (Theunissen et al., 2014). Reset pluripotent cells are transcriptionally distinct from conventional PSCs and more similar to mouse ESCs and human ICM (Davidson et al., 2015; Huang et al., 2014). They have increased mitochondrial respiratory activity and exhibit global DNA hypomethylation (Takashima et al., 2014), properties consistent with pre-implantation identity. Perhaps most persuasively, reset cells have acquired expression of, and functional dependency on, transcription factors KLF4 and TFCP2L1 constituting part of the core gene regulatory network of naive pluripotency in mouse ESCs (Dunn et al., 2014; Martello et al., 2013; Niwa et al., 2009; Ye et al., 2013) and are expressed in the human ICM but negligible in the primed PSC (Takashima et al., 2014).

In rodents functional equivalence of ESCs with naive epiblast can be demonstrated by blastocyst colonization and extensive multilineage contribution to chimeras. Such an assay is not feasible in human. An alternative indicator of developmental identity is propagation directly from naive epiblast cells, as for derivation of mouse ESCs (Boroviak et al., 2014; Brook and Gardner, 1997; Nichols et al., 2009). In human the standard process for establishing PSC lines from embryos entails explant outgrowth to form an epithelial structure (Pickering et al., 2003), the

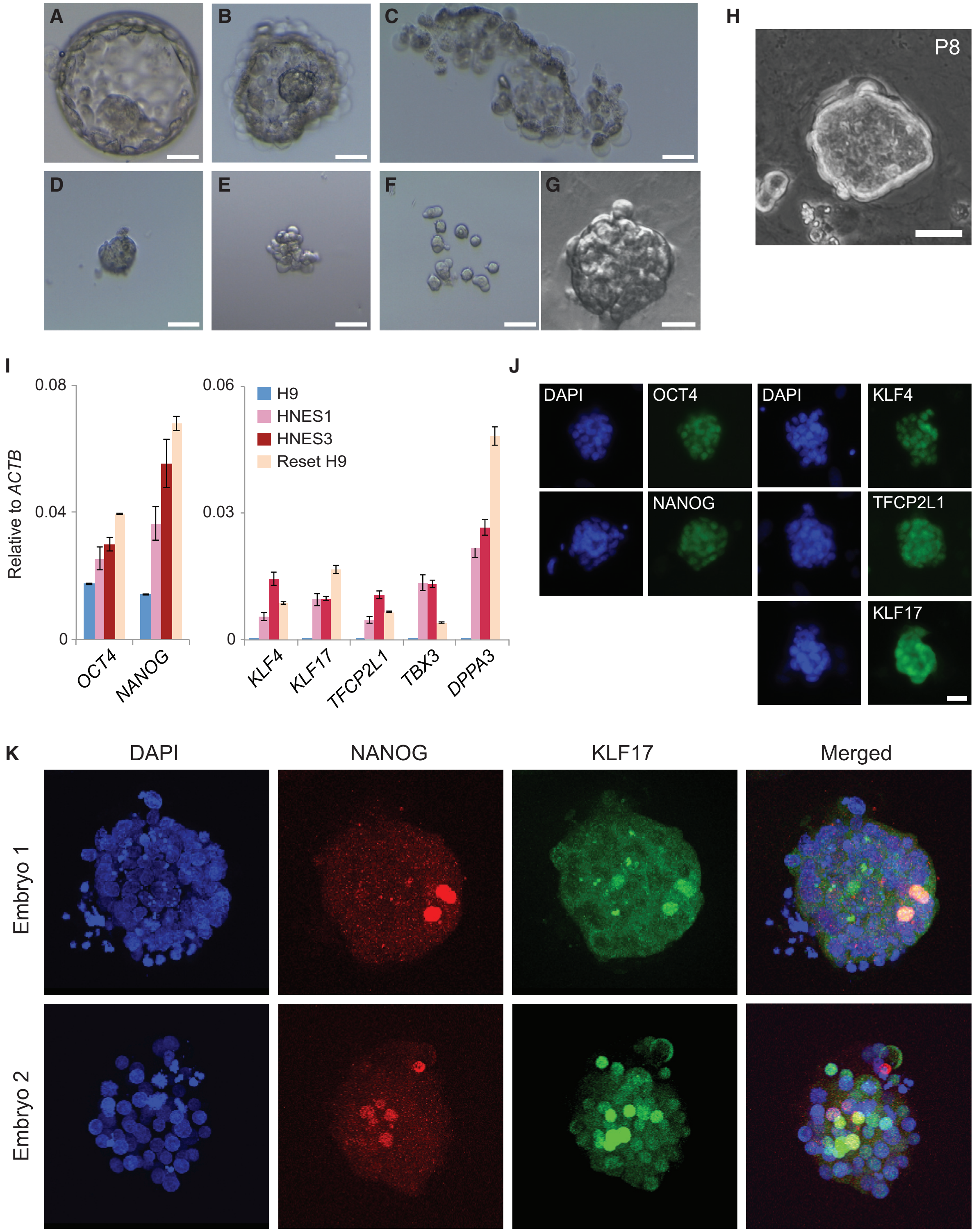

Figure 1. Cell Line Derivation from Dissociated Human Inner Cell Mass Cells

(A) Day-6 blastocyst.

(B) Trophoblast lysis.

(C) Discarded trophoblast.

(D) Isolated inner cell mass.

(legend continued on next page)

Table 1. Derivation of Naive Epiblast Stem Cell Lines

Experiment	Embryos Surviving Thaw	Blastocysts[a]	Dissociated ICMs	Cell Lines	Cumulative Passages
1	24	4	1	HNES1	P30
2	9	4	2	HNES2	P22
				HNES3	P29
3	20	4	4	HNES4[b]	P21
4	5	2	1	[c]	
Total	58	14	8	4	

[a]Embryos cavitated by day 6.
[b]Primary colonies lost in three cases associated with incubator humidity failure.
[c]Primary colonies emerged but failed to expand after five passages.

post-inner cell mass intermediate (PICMI) (O'Leary et al., 2012). This is thought to simulate development of the post-implantation embryonic disk (Van der Jeught et al., 2015), which may explain why derivative cell lines acquire characteristics of primed pluripotency. Naive pluripotency factors such as TFCP2L1 are downregulated during PICMI formation (O'Leary et al., 2012). We elected to test the ability of culture conditions that sustain human naive PSCs after resetting in vitro to support de novo derivation from dissociated human ICMs without PICMI transition.

RESULTS

Previous human embryo derivations of PSCs have been performed in the presence of fibroblast growth factor (FGF) and/or serum factors, conditions that support developmental progression. We avoided these and adopted the culture regime developed for human reset PSCs (Takashima et al., 2014), comprising serum-free N2B27 medium with LIF and t2i (inhibitors of GSK3 and MAPK/Erk signaling) plus the PKC inhibitor Gö6983. To safeguard viability of precious embryo cells, we added ascorbic acid and ROCK inhibitor (Y-27632), constituting t2iLGöY. Cultures were maintained throughout on fibroblast feeders in 5% O_2.

ICMs were isolated from blastocysts 6 days post-fertilization by immunosurgery (Solter and Knowles, 1975). Following dissociation, single cells or doublets were distributed on feeders in t2iLGöY. Up to half of the plated ICM cells formed compact colonies within 4–5 days (Figures 1A–1G), similar to mouse ESC primary colony formation (Nichols et al., 2009). For each embryo, colonies were manually picked, dissociated, and pooled. Replated cells proliferated (Figures 1H and S1A) and from a total of eight ICMs, four cell lines were established (Table 1) and provisionally termed human naive embryonic stem (HNES) cells.

HNES cells were expanded by passaging every 3–4 days, with ROCK inhibitor and ascorbic acid maintained throughout. HNES cells can be replated and maintained without ROCK inhibitor, albeit at lower efficiency, and propagated without ascorbic acid (Figures S1B–S1D). They can be cryopreserved and thawed with expected recovery efficiency using standard procedures. HNES1 cells exhibit a consistent 46XY karyotype with no abnormalities detected by G-banding (Figure S1E and Table S1), while HNES2 comprised both diploid and tetraploid cells on initial karyotyping but resolved to 46XY after flow sorting (Figures S1E and S1F). HNES3 is a mix of 46XX and cells with chr22 trisomy. HNES4 contains two isochromosomes of chromosome 12. Array comparative genomic hybridization at 200 kb genome-wide resolution confirmed lack of chromosomal abnormalities in HNES1. This line is described below with data from other lines where specified.

HNES cells expressed mRNAs for naive pluripotency markers *KLF4*, *TFCP2L1*, and *DPPA3*, along with elevated *NANOG* transcripts (Figure 1I) as seen in reset cells generated from conventional PSCs (Takashima et al., 2014). Immunostaining confirmed presence of NANOG, KLF4, TFCP2L1, and OCT4 (Figures 1J and S1G). Expression of *ESRRB* and *KLF2* was low in HNES cells, similar to reset cells. Both factors are also expressed at low levels in human and marmoset ICMs, indicating divergence between primates and rodents (Blakeley et al., 2015; Boroviak et al., 2015). Another Kruppel-like factor, KLF17, is observed at the transcript level in primate ICMs (Blakeley et al., 2015; Boroviak et al., 2015) and expressed in reset and HNES cells

(E) Decompacted ICM.
(F) Dissociated ICM.
(G) Primary stem cell clone grown from a single ICM cell.
(H) Colony at passage 8.
(I) qRT-PCR for pluripotency markers in HNES cells, conventional human PSCs (H9), and in vitro reset PSCs (Reset H9). Error bars indicate the SD of two independent reactions.
(J) Immunofluorescence of pluripotency markers in HNES1 cells.
(K) Immunofluorescence of KLF17 and NANOG in D6 human ICM cells.
Scale bars: 25 μm.

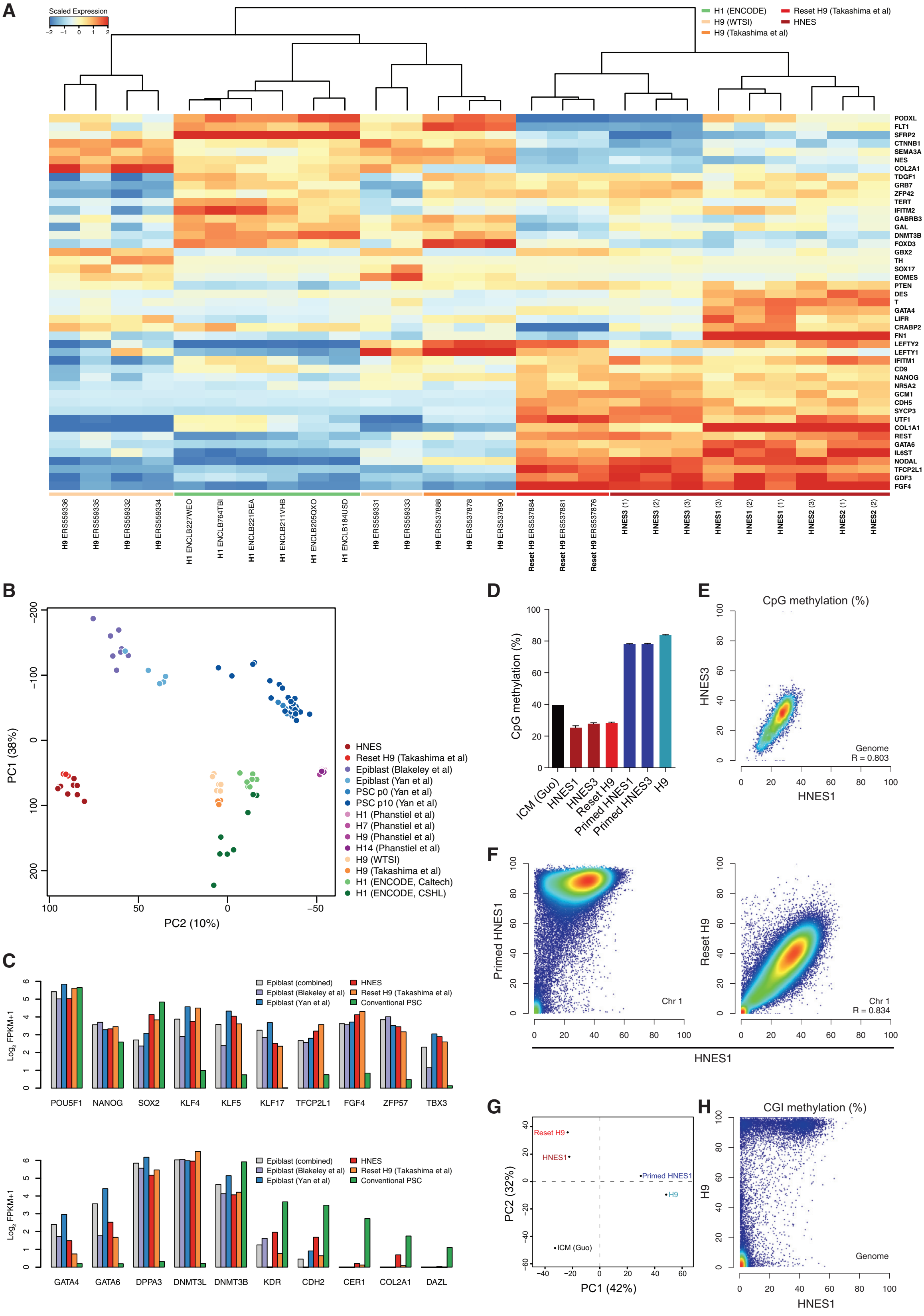

(legend on next page)

(Figure 1I). We detected KLF17 protein in HNES cells and human ICMs (Figures 1J and 1K).

Whole-transcriptome profiles were obtained by RNA-seq from replicate cultures of HNES1, HNES2, and HNES3. These were compared with reset and conventional human PSC datasets (Takashima et al., 2014) and to a wider panel of H1, H7, H9, and H14 data from the public domain. HNES cells feature a transcriptome distinct from other PSCs and close to the reset state (Figure 2A). They show consistent expression of naive pluripotency factors. Conventional PSCs exhibit wider variation in expression profiles with sporadic activation of naive factors such as *NANOG, ZFP42* (*REX1*), and *TFCP2L1*. HNES cells express a restricted complement of lineage markers compared with conventional PSCs. We performed principal component analysis (PCA), additionally incorporating published data (Blakeley et al., 2015; Yan et al., 2013) on human ICM cells and primary cultures generated by single-cell RNA-seq (Figure 2B). PC1 primarily discriminates between cells profiled by single-cell and bulk RNA-seq methods, suggesting a substantial contribution of global expression variance by sequencing protocol. Numerous transcripts present in conventional RNA-seq datasets register zero read counts in the single-cell libraries, in line with known detection limitations (Kharchenko et al., 2014). Biological replicates of the three HNES cells cluster together and adjacent to reset H9 cells. PC2 places HNES cells in relative proximity to the ICM cells and well separated from other PSCs. The degree of correspondence between HNES and embryo cells appears reasonable, considering the wider variation between samples profiled in the embryo studies, and that early ICM cells analyzed precede naive epiblast. Markers of naive pluripotency and lineage specification diverge between HNES and reset cells versus conventional PSC (Figure 2C).

Primed PSCs rely on anaerobic glycolysis with low mitochondrial respiration capacity (Zhou et al., 2012), whereas reset PSCs have active mitochondria and reduced glucose dependence (Takashima et al., 2014). We evaluated the capacity of HNES cells to form colonies in the presence of the competitive inhibitor of glycolysis, 2-deoxyglucose. Undifferentiated HNES cells readily formed colonies while primed HNES cells generated by passaging in FGF/KSR did not survive (Figure S2A). HNES cells also stained intensely with MitoProbe DiIC1, reflecting mitochondrial membrane potential (Figures S2B and S2C). Extracellular flux analysis indicated that HNES cells exhibit at least 2-fold higher respiratory capacity than primed cells (Figure S2D).

Global DNA hypomethylation is a distinguishing feature of mouse and human ICM cells (Guo et al., 2014; Smith et al., 2012), a property shared with naive ESCs (Ficz et al., 2013; Habibi et al., 2013; Leitch et al., 2013) and reset human PSCs (Takashima et al., 2014). Immunostaining for 5-methylcytosine (5mC) is fainter in HNES cell nuclei compared with primed HNES cells (Figure S3A). Like reset PSCs, HNES cells show appreciable expression of TET1 and downregulation of de novo methyltransferase DNMT3B (Figures S3B and S3C). We performed whole-genome bisulfite sequencing on two HNES lines and their primed derivatives. Analysis confirmed genome-wide hypomethylation in male and female HNES cells, similar to levels of 25%–40% observed in human ICM and in contrast to >70% CpG methylation in conventional PSCs and primed HNES cells (Figure 2D). Both HNES lines showed extensive overlap in the distribution of CpG methylation sites (Figure 2E), with substantial hypomethylation compared with primed HNES cells (Figure 2F). The methylomes of HNES and reset H9 cells are very similar, suggesting that the epigenetic state of conventional human PSCs can be accurately and consistently reprogrammed. We analyzed methylation levels of CpG islands (CGIs) and performed PCA, revealing clustering of HNES with reset H9 cells and conventional human PSCs (H9) with primed HNES cells (Figure 2G). PC1 captured most of the variation (42%), indicating high resemblance between HNES cells and human ICMs (Guo et al., 2014). Comparisons of CGI

Figure 2. Transcriptome and Methylome Analyses

(A) Clustered expression data from HNES cells, and reset and conventional human PSCs for a panel of pluripotency and lineage markers selected by the International Stem Cell Initiative (Adewumi et al., 2007). Displayed are $\log_2$ FPKM values (fragments per kilobase of exon per million reads mapped) scaled by the mean expression of each gene across samples. Published data are labeled with sample accession codes.

(B) PCA of HNES cells, and reset and conventional PSCs with single-cell RNA-seq data from early human ICMs (Blakeley et al., 2015; Yan et al., 2013) and PSC explants. Embryo single-cell samples are those assigned an epiblast identity in the respective studies.

(C) Pluripotency and lineage marker expression in human ICM, HNES cells, and reset and conventional PSC lines.

(D) Proportion of whole-genome CpG methylation measured by bisulfite sequencing (BS-seq) analysis from three biological replicates. Error bars indicate the SD of three biological replicates.

(E) Comparison of global methylation in HNES1 (male) and HNES3 (female) cells by averaging CpG methylation levels over 500-kb windows.

(F) Comparisons of CpG methylation in HNES1 cells and primed derivatives, and reset H9 and ICM cells.

(G) PCA of mean CpG island methylation.

(H) CGI methylation in HNES1 and conventional PSCs.

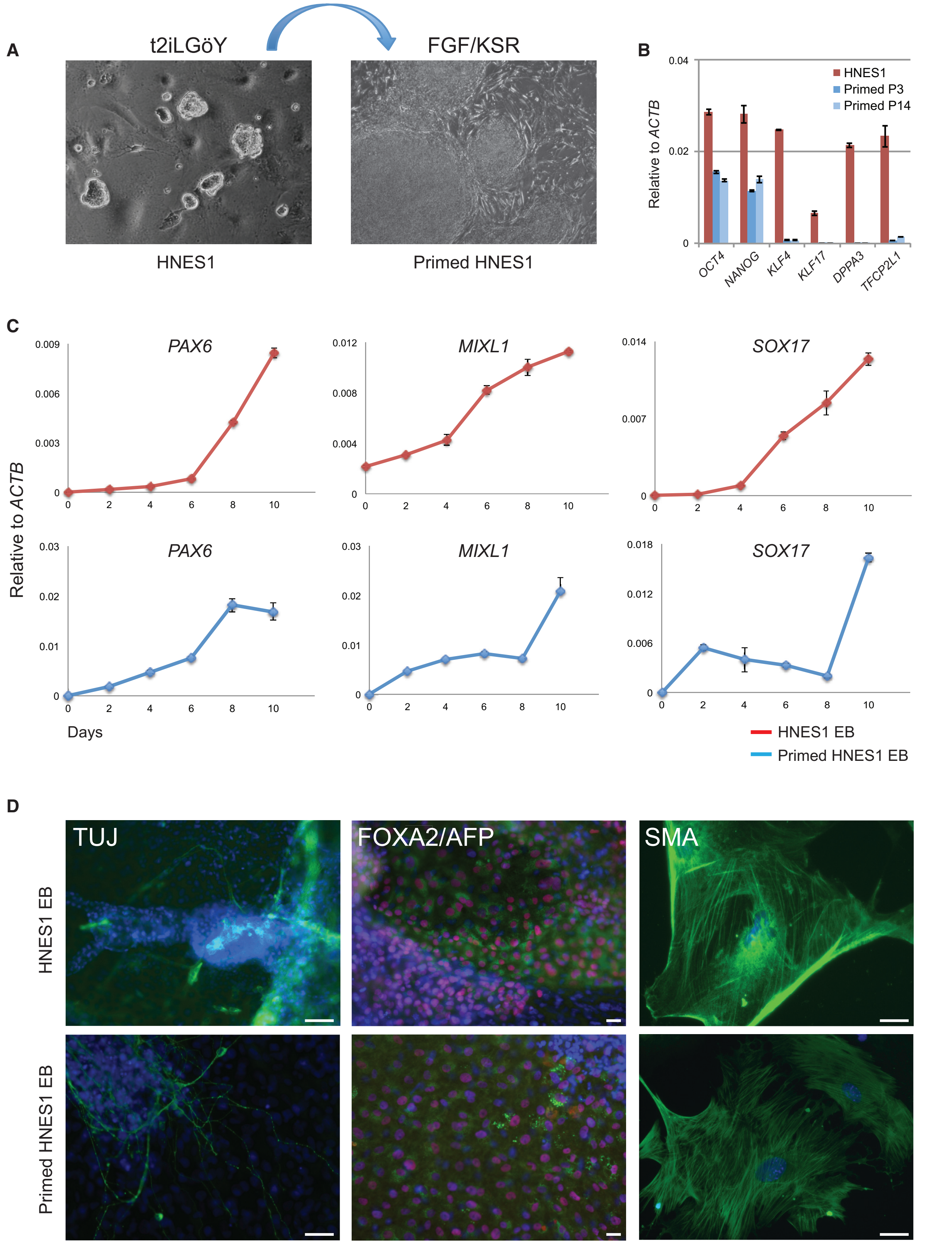

(legend on next page)

methylation in HNES and H9 cells (Figure 2H) showed that the majority of CGIs are hypomethylated in both HNES cells and conventional PSCs, while many CGIs gain methylation in primed cells. Only a subset of CGIs is methylated in both conditions. These data highlight similarity between HNES and human ICM methylomes and show that conventional human PSCs have gained methylation at a number of CGIs when compared with HNES cells.

We transferred HNES cells to conventional PSC culture medium containing FGF/KSR and lacking inhibitors. After one passage the domed colonies of HNES cells assumed flattened epithelial morphology, and after two passages resembled conventional PSC (Figure 3A). During this transition *OCT4* and *NANOG* were reduced, and naive markers, including *KLF17*, were extinguished (Figures 3B, S4A, and S4B).

We assessed whether HNES cells can undergo multilineage differentiation by generating embryoid bodies directly from naive and primed HNES cells. In both cases early lineage markers *PAX6*, *MIXL1*, and *SOX17* were upregulated (Figure 3C). Outgrowths from plated embryoid bodies displayed TuJ1-positive neuronal, FOXA2/AFP double-positive endoderm, and smooth muscle actin-positive cells (Figure 3D). We also applied a protocol for cardiomyocyte differentiation (van den Berg et al., 2016) to primed HNES cells and observed multiple regions of spontaneous contraction after 12 days (Movie S1). Cardiomyocyte identity was confirmed by expression of surface markers VCAM-1 and CD172a (SIRPα) (Figure S4C).

DISCUSSION

Hitherto, stem cell derivations from human embryos have yielded cells with features distinct from rodent ESCs and more similar to mouse post-implantation epiblast-derived stem cells (Brons et al., 2007; Tesar et al., 2007). This may be because the culture conditions used were inadequate to sustain naive pluripotency in the face of stimuli for developmental progression emanating from extraembryonic endoderm (Brook and Gardner, 1997) in ICM explants and/or provided by FGF and serum factors. Even for derivations commencing from single blastomeres, a blastocyst-like structure develops, followed by ICM outgrowth prior to cell line derivation (Taei et al., 2013). We show that after dissociation of the ICM to separate epiblast and primitive endoderm, stem cell colonies emerge directly in the presence of inhibitors of MAPK/Erk, GSK3, and PKC. Resulting HNES cell lines can be propagated by enzymatic dissociation to single cells, retain chromosomal integrity over many passages, exhibit features diagnostic of naive pluripotency, and are capable of multilineage differentiation.

Conventional human PSC cultures are heterogeneous, potentially comprising complex hierarchies (Davidson et al., 2015; Enver et al., 2009; Hough et al., 2014). Furthermore, pluripotency is an inherently plastic stage of development. It is unsurprising, therefore, that PSCs can adjust to alternative culture conditions with shifts in morphology and gene expression. Without objective criteria, these may be misinterpreted as a change in developmental status rather than accommodation to culture. In contrast, global transcriptome, metabolic properties, and DNA hypomethylation features align HNES cells with reset PSCs and distinguish them from conventional human PSCs. Of particular significance, HNES cells and reset PSCs express the naive pluripotency factors *KLF4*, *TFCP2L1*, *TBX3*, and *NANOG* found in the primate ICMs and functional in mouse ESC self-renewal. Additionally they express *KLF17*, which might compensate for lower expression of *KLF2*. Apart from NANOG, these factors are expressed at low levels or not at all in conventional human PSCs, including those variants purported to be naive by other criteria. We have shown that the reset PSC state is dependent on both KLF4 and TFCP2L1 (Takashima et al., 2014).

The naive gene regulatory network is not fully conserved between mouse and human. Absence of ESRRB marks a substantial distinction. Mouse ESCs can be maintained after deletion of *Esrrb* but are less stable (Martello et al., 2012). Lack of ESRRB may therefore render human naive PSC propagation inherently more demanding. Nonetheless, culture refinements and replacement of feeders with a defined substrate may be anticipated to facilitate their handling and possibly attainment of a ground state.

In summary, these findings suggest that it is possible to suspend human developmental progression at the pre-implantation epiblast phase and propagate a self-renewing pluripotent state analogous to mouse ESCs (Boroviak et al., 2014; Brook and Gardner, 1997). Derivation of equivalent cell lines from non-human primates and formation of high-contribution chimeras would provide further

Figure 3. Differentiation

(A) Colonies of naive HNES1 cells in t2iLGöY and primed HNES1 cells after 12 passages in FGF/KSR.
(B) qRT-PCR analysis of naive marker expression in naive HNES1 cells and derivatives after three passages in FGF/KSR. Error bars indicate the SD of two independent reactions.
(C) qRT-PCR analysis of embryoid bodies formed from HNES1 and primed HNES1 cells. Error bars indicate the SD two independent reactions.
(D) Immunofluorescence of embryoid body outgrowths: TuJ1, β-III tubulin; AFP, α-fetoprotein; SMA, α-smooth muscle actin (green); FOXA2 (red). Nuclei (DAPI; blue). Scale bars, 100 μm.

validation. However, our results support the case for naive pluripotency in human development and may reconcile the long-running debate about the difference between PSCs from mice and men.

EXPERIMENTAL PROCEDURES

Embryo Manipulation

Supernumerary frozen human embryos were donated with informed consent under license from the UK HFEA. Embryos were thawed using EmbryoThaw medium (FertiPro) and cultured in drops of pre-equilibrated medium (Origio): EmbryoAssist for 1–8 cell stage (days 0–2), and BlastAssist for 8 cell stage to blastocyst (days 3–6) under embryo-tested mineral oil (Sigma). Expanded blastocysts (day 6) were subjected to immunosurgery (Pickering et al., 2005) to isolate ICMs using anti-human serum (Sigma). ICMs were treated with Accutase (Sigma or Gibco) for 5–10 min, and placed in a drop of medium for mechanical separation using a finely drawn Pasteur pipette. ICM cells were scattered onto mitotically inactivated (irradiated) murine embryonic fibroblasts (MEFs). Immunostaining was performed as described by Takashima et al. (2014).

Naive Stem Cell Culture

Cells were propagated in modified N2B27 medium supplemented with PD0325901 (1 μM, prepared in-house), CHIR99021 (1 μM, prepared in-house), Gö6983 (2.5 μM, Sigma-Aldrich), rho-associated kinase inhibitor (Y-27632) (10 μM, Calbiochem), human LIF (10 ng/ml, prepared in-house), and ascorbic acid (250 μM, Sigma). N2B27 medium (1 l) comprised 490 ml of DMEM/F12 (Life Technologies), 490 ml of Neurobasal (Life Technologies), 10 ml of B27 (Life Technologies), 5 ml of N2 (prepared in-house), 10 μg/ml insulin (Sigma), 2 mM L-glutamine (Life Technologies), and 0.1 mM 2-mercaptoethanol (Sigma). N2 contains 100 μg/ml apo-transferrin (eBioscience, ABC2553), 3 μM sodium selenite (Sigma), 1.6 mg/ml putrescine (Sigma), and 2 μg/ml progesterone (Sigma) in DMEM/F12 (Life Technologies). Primary colonies and nascent cell lines were passaged manually as described above for ICMs. Established cells were passaged either manually with Accutase (Life Technologies) dissociation reagent or as a pool using TrypLE Express (Life Technologies). Cells were cultured in 5% O_2 and 7% CO_2 in a humidified incubator at 37°C. Cells were frozen in 50% t2iLGöY medium with 40% serum and 10% DMSO.

Conversion to Primed Pluripotency

HNES cells were seeded on MEFs in t2iLGöY for 24 hr, then transferred into FGF/KSR medium for 7–10 days before passaging with TrypLE Express. Y-27632 was added for the first passage. Thereafter cells were passaged as clusters using collagenase/dispase (Roche). FGF/KSR medium comprised 20% KnockOut Serum Replacement (Invitrogen), 1× non-essential amino acids (Invitrogen), 2 mM L-glutamine (Invitrogen), 100 μM 2-mercaptoethanol (Sigma), 10 ng/ml FGF2 (prepared in-house), and DMEM/F-12 basal medium (Sigma-Aldrich). Established primed HNES cultures can also be maintained in mTeSR1 or E8 media (StemCell Technologies) on Matrigel.

In Vitro Differentiation

HNES cells or primed derivatives were dissociated with TrypLE Express and placed in PrimeSurface 96V cell plates (Sumitomo Bakelite MS-9096V) at a density of 4,000–5,000 cells per well in medium containing 20% KSR. Y-27632 was added during the first 24 hr of aggregation. At day 7 aggregates were plated on gelatin in 20% FBS.

Cardiomyocyte differentiation was performed as described by van den Berg et al. (2016).

ACCESSION NUMBERS

Data from this study are available in ArrayExpress: E-MTAB-4461 (RNA-seq), E-MTAB-4462 (BS-seq) and E-MTAB-4463 (Affymetrix).

SUPPLEMENTAL INFORMATION

Supplemental Information includes Supplemental Experimental Procedures, four figures, seven tables, and one movie and can be found with this article online at http://dx.doi.org/10.1016/j.stemcr.2016.02.005.

AUTHOR CONTRIBUTIONS

J.N., A.S., and G.G. planned the study; G.G., F.v.M., F.S., Y.C., P.B., and J.N. carried out experiments and analyses; P.B. performed RNA sequencing and bioinformatics; W.R. supervised methylome studies; J.N., A.S., P.B., and G.G. prepared the manuscript in consultation with all authors.

ACKNOWLEDGMENTS

We are grateful to Sally Marchant, Karen Thompson, and the staff and patients at the Centres for Reproductive Medicine at Barts and Leeds hospitals. We thank Ken Jones, James Clarke, Rosalind Drummond, Agata Kurowski, and Sally Lees for valuable technical assistance, Christine Mummery for the cardiomyocyte protocol, and Thorsten Boroviak for helpful comments. This work was supported by the Medical Research Council, Biotechnology and Biological Sciences Research Council, Swiss National Science Foundation (SNF)/Novartis SNF (F.v.M.), and core funding to the Cambridge Stem Cell Institute from the Wellcome Trust and Medical Research Council. A.S. is a Medical Research Council Professor. A.S. and G.G. are inventors on a patent application relating to naive human PSCs filed by the University of Cambridge.

Received: August 13, 2015
Revised: February 7, 2016
Accepted: February 8, 2016
Published: March 3, 2016

REFERENCES

Adewumi, O., Aflatoonian, B., Ahrlund-Richter, L., Amit, M., Andrews, P.W., Beighton, G., Bello, P.A., Benvenisty, N., Berry, L.S., Bevan, S., et al. (2007). Characterization of human embryonic stem cell lines by the International Stem Cell Initiative. Nat. Biotechnol. *25*, 803–816.

Blakeley, P., Fogarty, N.M., Del Valle, I., Wamaitha, S.E., Hu, T.X., Elder, K., Snell, P., Christie, L., Robson, P., and Niakan, K.K.

(2015). Defining the three cell lineages of the human blastocyst by single-cell RNA-seq. Development *142*, 3151–3165.

Boroviak, T., Loos, R., Bertone, P., Smith, A., and Nichols, J. (2014). The ability of inner-cell-mass cells to self-renew as embryonic stem cells is acquired following epiblast specification. Nat. Cell Biol. *16*, 516–528.

Boroviak, T., Loos, R., Lombard, P., Okahara, J., Behr, R., Sasaki, E., Nichols, J., Smith, A., and Bertone, P. (2015). Lineage-specific profiling delineates the emergence and progression of naive pluripotency in mammalian embryogenesis. Dev. Cell *35*, 366–382.

Brons, I.G., Smithers, L.E., Trotter, M.W., Rugg-Gunn, P., Sun, B., Chuva de Sousa Lopes, S.M., Howlett, S.K., Clarkson, A., Ahrlund-Richter, L., Pedersen, R.A., et al. (2007). Derivation of pluripotent epiblast stem cells from mammalian embryos. Nature *448*, 191–195.

Brook, F.A., and Gardner, R.L. (1997). The origin and efficient derivation of embryonic stem cells in the mouse. Proc. Natl. Acad. Sci. USA *94*, 5709–5712.

Davidson, K.C., Mason, E.A., and Pera, M.F. (2015). The pluripotent state in mouse and human. Development *142*, 3090–3099.

Dunn, S.J., Martello, G., Yordanov, B., Emmott, S., and Smith, A.G. (2014). Defining an essential transcription factor program for naive pluripotency. Science *344*, 1156–1160.

Dutta, D., Ray, S., Home, P., Larson, M., Wolfe, M.W., and Paul, S. (2011). Self-renewal versus lineage commitment of embryonic stem cells: protein kinase C signaling shifts the balance. Stem Cells *29*, 618–628.

Enver, T., Pera, M., Peterson, C., and Andrews, P.W. (2009). Stem cell states, fates, and the rules of attraction. Cell Stem Cell *4*, 387–397.

Ficz, G., Hore, T.A., Santos, F., Lee, H.J., Dean, W., Arand, J., Krueger, F., Oxley, D., Paul, Y.L., Walter, J., et al. (2013). FGF signaling inhibition in ESCs drives rapid genome-wide demethylation to the epigenetic ground state of pluripotency. Cell Stem Cell *13*, 351–359.

Guo, H., Zhu, P., Yan, L., Li, R., Hu, B., Lian, Y., Yan, J., Ren, X., Lin, S., Li, J., et al. (2014). The DNA methylation landscape of human early embryos. Nature *511*, 606–610.

Habibi, E., Brinkman, A.B., Arand, J., Kroeze, L.I., Kerstens, H.H., Matarese, F., Lepikhov, K., Gut, M., Brun-Heath, I., Hubner, N.C., et al. (2013). Whole-genome bisulfite sequencing of two distinct interconvertible DNA methylomes of mouse embryonic stem cells. Cell Stem Cell *13*, 360–369.

Hackett, J.A., and Surani, M.A. (2014). Regulatory principles of pluripotency: from the ground state up. Cell Stem Cell *15*, 416–430.

Hough, S.R., Thornton, M., Mason, E., Mar, J.C., Wells, C.A., and Pera, M.F. (2014). Single-cell gene expression profiles define self-renewing, pluripotent, and lineage primed states of human pluripotent stem cells. Stem Cell Rep. *2*, 881–895.

Huang, K., Maruyama, T., and Fan, G. (2014). The naive state of human pluripotent stem cells: a synthesis of stem cell and preimplantation embryo transcriptome analyses. Cell Stem Cell *15*, 410–415.

Kharchenko, P.V., Silberstein, L., and Scadden, D.T. (2014). Bayesian approach to single-cell differential expression analysis. Nat. Methods *11*, 740–742.

Leitch, H.G., McEwen, K.R., Turp, A., Encheva, V., Carroll, T., Grabole, N., Mansfield, W., Nashun, B., Knezovich, J.G., Smith, A., et al. (2013). Naive pluripotency is associated with global DNA hypomethylation. Nat. Struct. Mol. Biol. *20*, 311–316.

Martello, G., and Smith, A. (2014). The nature of embryonic stem cells. Annu. Rev. Cell Dev. Biol. *30*, 647–675.

Martello, G., Sugimoto, T., Diamanti, E., Joshi, A., Hannah, R., Ohtsuka, S., Gottgens, B., Niwa, H., and Smith, A. (2012). Esrrb is a pivotal target of the Gsk3/Tcf3 axis regulating embryonic stem cell self-renewal. Cell Stem Cell *11*, 491–504.

Martello, G., Bertone, P., and Smith, A. (2013). Identification of the missing pluripotency mediator downstream of leukaemia inhibitory factor. EMBO J. *32*, 2561–2574.

Nichols, J., and Smith, A. (2009). Naive and primed pluripotent states. Cell Stem Cell *4*, 487–492.

Nichols, J., Silva, J., Roode, M., and Smith, A. (2009). Suppression of Erk signalling promotes ground state pluripotency in the mouse embryo. Development *136*, 3215–3222.

Niwa, H., Ogawa, K., Shimosato, D., and Adachi, K. (2009). A parallel circuit of LIF signalling pathways maintains pluripotency of mouse ES cells. Nature *460*, 118–122.

O'Leary, T., Heindryckx, B., Lierman, S., van Bruggen, D., Goeman, J.J., Vandewoestyne, M., Deforce, D., de Sousa Lopes, S.M., and De Sutter, P. (2012). Tracking the progression of the human inner cell mass during embryonic stem cell derivation. Nat. Biotechnol. *30*, 278–282.

Pickering, S.J., Braude, P.R., Patel, M., Burns, C.J., Trussler, J., Bolton, V., and Minger, S. (2003). Preimplantation genetic diagnosis as a novel source of embryos for stem cell research. Reprod. Biomed. Online *7*, 353–364.

Pickering, S.J., Minger, S.L., Patel, M., Taylor, H., Black, C., Burns, C.J., Ekonomou, A., and Braude, P.R. (2005). Generation of a human embryonic stem cell line encoding the cystic fibrosis mutation deltaF508, using preimplantation genetic diagnosis. Reprod. Biomed. Online *10*, 390–397.

Rossant, J. (2015). Mouse and human blastocyst-derived stem cells: vive les differences. Development *142*, 9–12.

Smith, Z.D., Chan, M.M., Mikkelsen, T.S., Gu, H., Gnirke, A., Regev, A., and Meissner, A. (2012). A unique regulatory phase of DNA methylation in the early mammalian embryo. Nature *484*, 339–344.

Solter, D., and Knowles, B.B. (1975). Immunosurgery of mouse blastocyst. Proc. Natl. Acad. Sci. USA *72*, 5099–5102.

Taei, A., Hassani, S.-N., Eftekhari-Yazdi, P., Rezazadeh Valojerdi, M., Nokhbatolfoghahai, M., Masoudi, N.-S., Pakzad, M., Gourabi, H., and Baharvand, H. (2013). Enhanced generation of human embryonic stem cells from single blastomeres of fair and poor-quality cleavage embryos via inhibition of glycogen synthase kinase β and Rho-associated kinase signaling. Hum. Reprod. *28*, 2661–2671.

Takashima, Y., Guo, G., Loos, R., Nichols, J., Ficz, G., Krueger, F., Oxley, D., Santos, F., Clarke, J., Mansfield, W., et al. (2014).

Resetting transcription factor control circuitry toward ground-state pluripotency in human. Cell *158*, 1254–1269.

Tesar, P.J., Chenoweth, J.G., Brook, F.A., Davies, T.J., Evans, E.P., Mack, D.L., Gardner, R.L., and McKay, R.D. (2007). New cell lines from mouse epiblast share defining features with human embryonic stem cells. Nature *448*, 196–199.

Theunissen, T.W., Powell, B.E., Wang, H., Mitalipova, M., Faddah, D.A., Reddy, J., Fan, Z.P., Maetzel, D., Ganz, K., Shi, L., et al. (2014). Systematic identification of culture conditions for induction and maintenance of naive human pluripotency. Cell Stem Cell *15*, 471–487.

van den Berg, C.W., Elliott, D.A., Braam, S.R., Mummery, C.L., and Davis, R.P. (2016). Differentiation of human pluripotent stem cells to cardiomyocytes under defined conditions. Methods Mol. Biol. *1353*, 163–180.

Van der Jeught, M., O'Leary, T., Duggal, G., De Sutter, P., Chuva de Sousa Lopes, S., and Heindryckx, B. (2015). The post-inner cell mass intermediate: implications for stem cell biology and assisted reproductive technology. Hum. Reprod. Update *21*, 616–626.

Yan, L., Yang, M., Guo, H., Yang, L., Wu, J., Li, R., Liu, P., Lian, Y., Zheng, X., Yan, J., et al. (2013). Single-cell RNA-Seq profiling of human preimplantation embryos and embryonic stem cells. Nat. Struct. Mol. Biol. *20*, 1131–1139.

Ye, S., Li, P., Tong, C., and Ying, Q.L. (2013). Embryonic stem cell self-renewal pathways converge on the transcription factor Tfcp2l1. EMBO J. *32*, 2548–2560.

Ying, Q.L., Wray, J., Nichols, J., Batlle-Morera, L., Doble, B., Woodgett, J., Cohen, P., and Smith, A. (2008). The ground state of embryonic stem cell self-renewal. Nature *453*, 519–523.

Zhou, W., Choi, M., Margineantu, D., Margaretha, L., Hesson, J., Cavanaugh, C., Blau, C.A., Horwitz, M.S., Hockenbery, D., Ware, C., et al. (2012). HIF1alpha induced switch from bivalent to exclusively glycolytic metabolism during ESC-to-EpiSC/hESC transition. EMBO J. *31*, 2103–2116.

Stem Cell Reports, Volume *6*

Supplemental Information

Naive Pluripotent Stem Cells Derived Directly from Isolated Cells of the Human Inner Cell Mass

Ge Guo, Ferdinand von Meyenn, Fatima Santos, Yaoyao Chen, Wolf Reik, Paul Bertone, Austin Smith, and Jennifer Nichols

Supplemental Figures

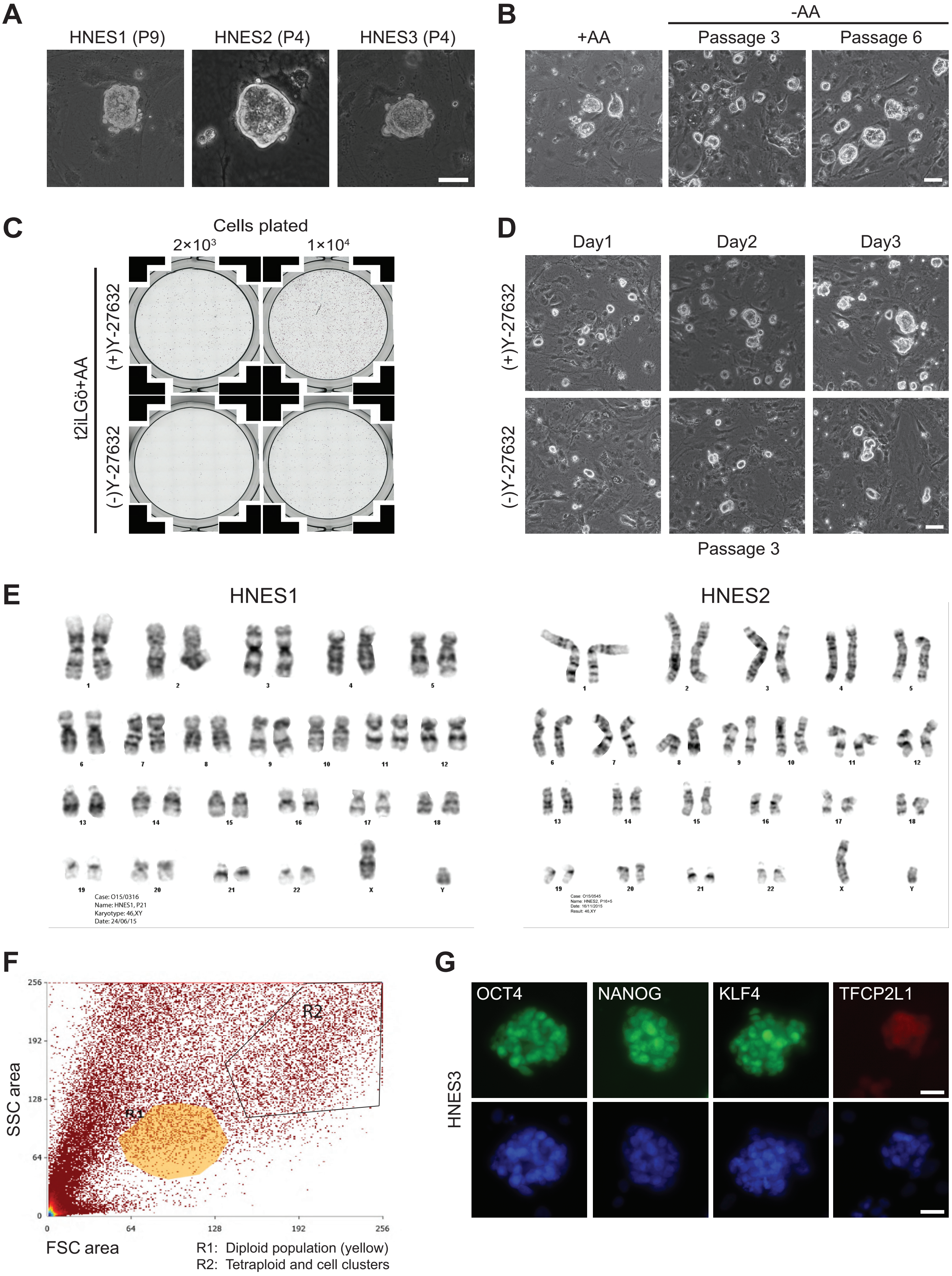

Figure S1, related to Figure 1: (A) Colonies of HNES cells. Passage numbers are shown in brackets. (B) HNES1 cells in t2iLGö with or without ascorbic acid (AA). (C) HNES1 colony formation assay in t2iLGöY or after withdrawal of ROCK inhibitor (Y-27632). (D) HNES1 cells expanding after three passages with or without ROCK inhibitor. (E) Karyotype analysis of HNES1 and HNES2 cells by Giemsa staining. (F) Density plot showing HNES2 diploid cells (R1) sorted by flow cytometry. (G) Immunofluorescence staining of pluripotency markers. Scale bar, 25 µm.

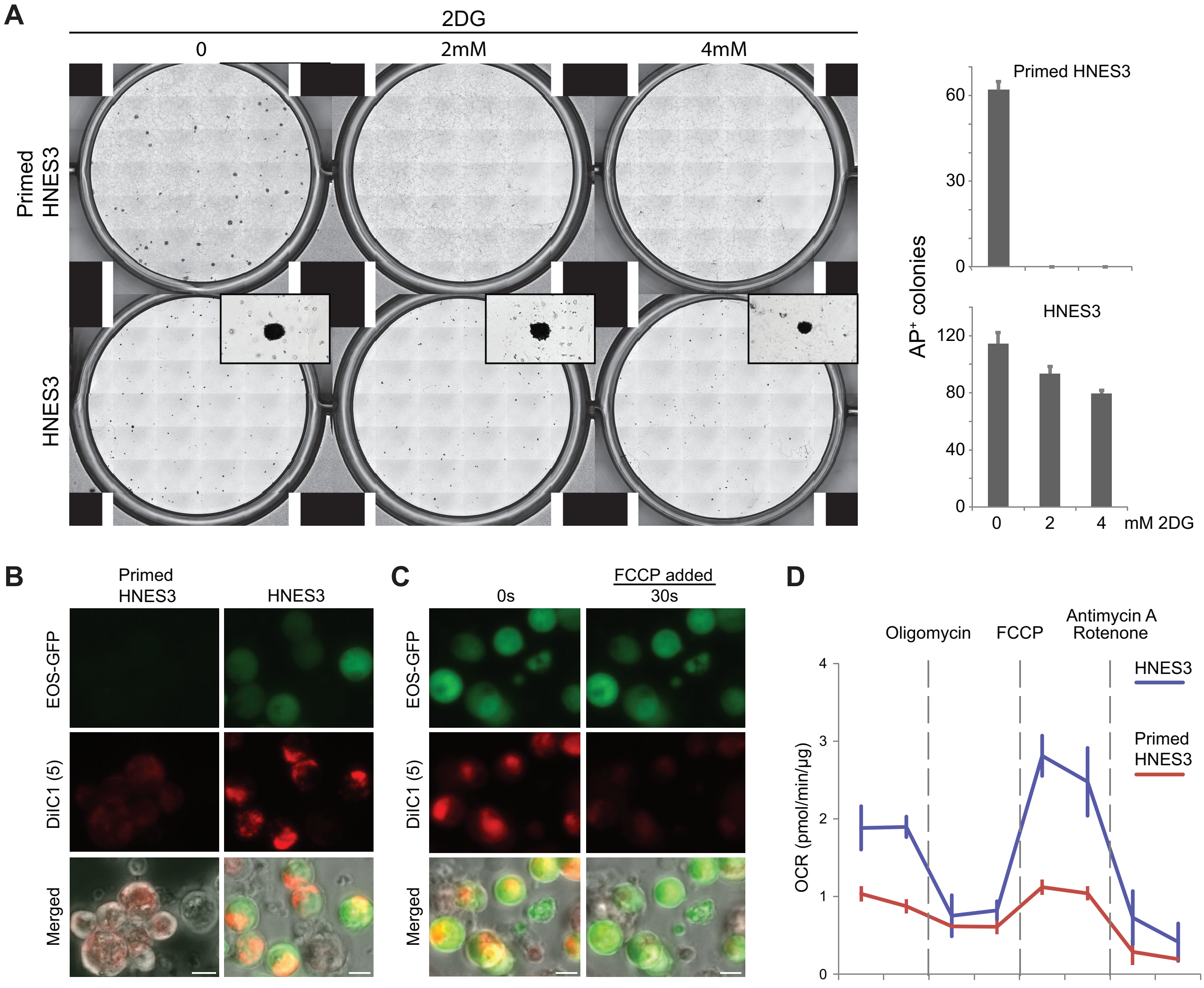

Figure S2, related to Results: (A) Colony formation assay in culture conditions supplemented with the indicated concentrations of 2-deoxy-D-glucose (2DG). Quantification, right. Error bars indicate standard deviation (s.d.) of two replicates. (B) Mitochondrial activity staining using mitochondrial membrane potential-dependent MitoProbe DilC1 (5). (C) Stain intensity decreases after cells are treated with the mitochondrial uncoupling reagent carbonyl cyanide-4-(trifluoromethoxy)phenylhydrazone (FCCP), confirming signal specificity. (D) Oxygen consumption rate (OCR) measured by the XF Cell Mito Stress Test assay provides a complete mitochondrial profile (see Extended Experimental Procedures). HNES cells exhibit higher OCR relative to primed PSC, particularly at elevated mitochondrial respiration following exposure to FCCP. Error bars indicate s.d. of five replicates.

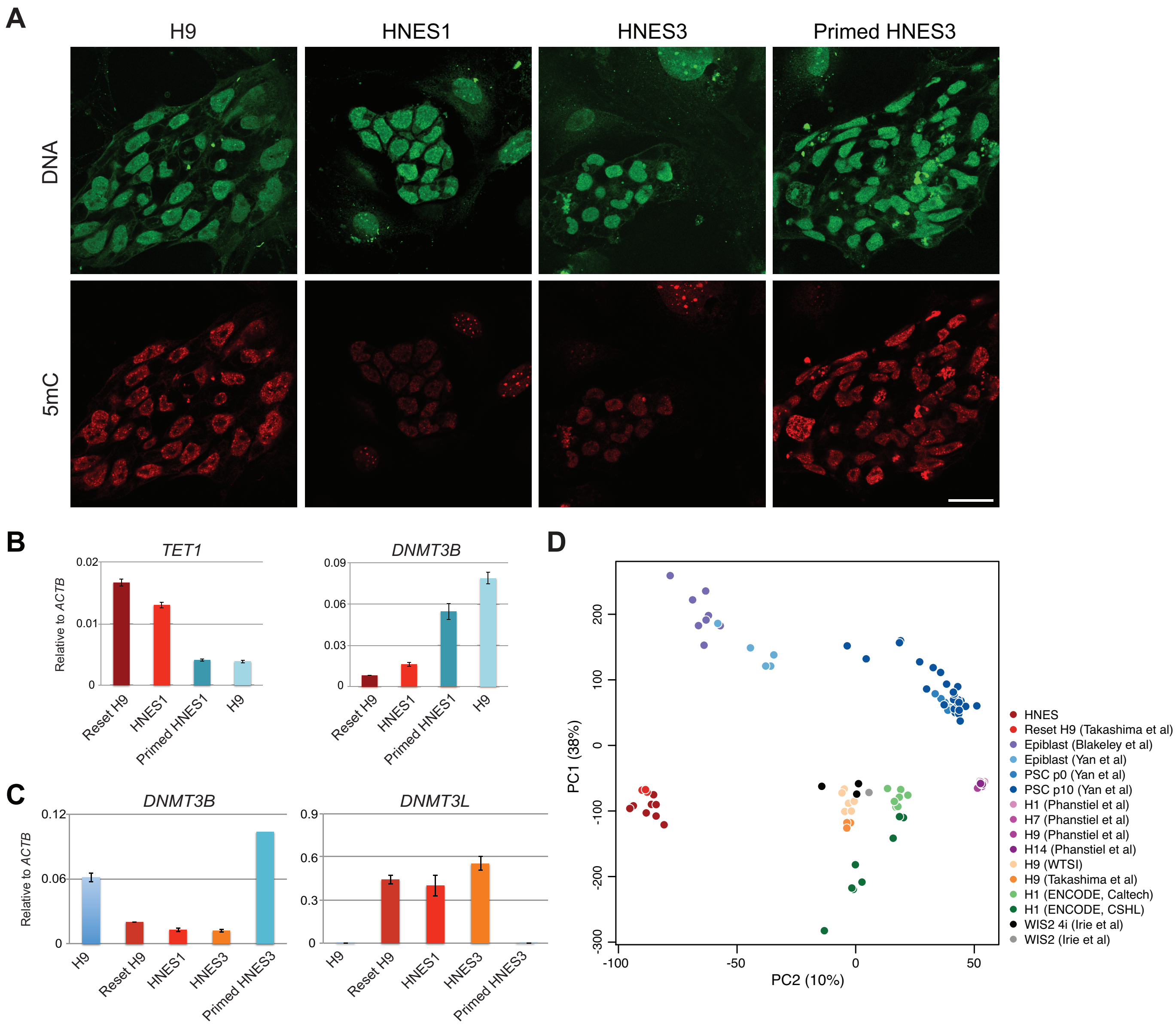

Figure S3, related to Figure 2: (A) Immunofluorescence staining of 5mC (red). DNA was counterstained with YOYO-1 (green). Scale bar, 20 µm. (B, C) qRT-PCR analysis of human pluripotent cell lines for TET1 and DNA methyltransferases. Error bars indicate s.d. of two independent reactions. (D) Principal component analysis of samples represented in Figure 2B including WIS2 human PSC cultured in conventional and modified NHSM (4i) conditions (Irie et al., 2015).

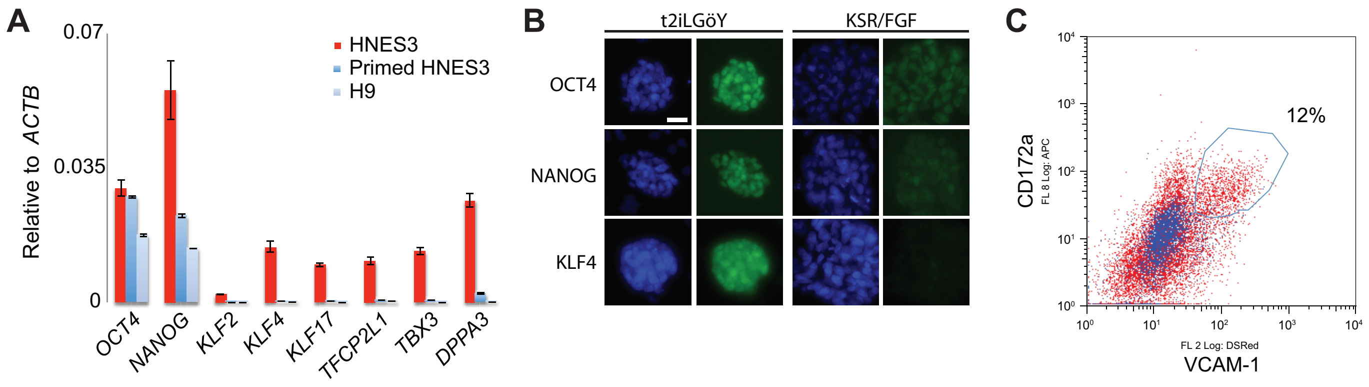

Figure S4, related to Figure 3: (A) qRT-PCR analysis showing reduced expression of naïve pluripotency markers in primed HNES3 and H9 cultures compared to naïve HNES3 cells. Error bars indicate s.d. of two independent reactions. (B) Immunofluorescence staining showing loss of expression of KLF4 in primed HNES3 cells. Scale bar, 25µm. (C) Flow cytometry analysis to isolate the CD172a and VCAM-1 double-positive cardiomyocyte population following differentiation for 12 days.

Supplemental Tables

Table S1, related to Table 1: Chromosomal analysis of HNES cell lines.

Cell line	Passage	Karyotype
HNES1*	P11<br>P21	46 XY (20/20)<br>46 XY (11/11)
HNES2	P6<br>P16+5	46 XY (10/30); tetraploid-like (20/30)<br>46 XY (20/20)
HNES3	P18	46 XX (6/20); 47 XX (14/20),+22^
HNES4	P20	48 XY, +i(12)(P10),+i(12)(P10)

* HNES1 cells were also examined by array CGH after 14 passages cultured in t2iLGöY and a further 8 passages in FGF/KSR. This analysis confirmed a 46 XY chromosome complement with no detectable abnormalities.

^ Heterogeneity for trisomy 22 observed in HNES3 may reflect mosaicism in the original ICM. Trisomy 22 is among the most frequently occurring chromosomal abnormalities causing spontaneous abortion during the first trimester (Nagaishi et al., 2004; Sasabe et al., 1999).

Table S2, related to Figures 1, 3 and S4: TaqMan qPCR assays.

Gene	TaqMan assay
ACTB	Hs01060665_g1
POU5F1	Hs01654807_s1
NANOG	Hs02387400_g1
KLF17	Hs00703004_s1
KLF4	Hs00358836_m1
KLF2	Hs00360439_g1
DPPA3	Hs01931905_g1
TFCP2L1	Hs00232708_m1
TBX3	Hs00195612_m1
DNMT3B	Hs00171876_m1
DNMT3L	Hs01081364_m1
TET1	Hs00286756_m1
FOXA2	Hs00232764_m1
PAX6	Hs00240871_m1
SOX17	Hs00751752_s1
MIXL1	Hs00430824_g1

Table S3, related to Figures 1, 3, S1, S3 and S4: Primary antibodies used in this study.

Antibody	Vendor	Number	Dilution
OCT3/4	Santa Cruz	sc-5279	1:200
NANOG	eBioscience	14-5769-82	1:200
KLF4	Santa Cruz	sc-20691	1:400
TFCP2L1	R&D	AF5726	1:500
TUJ1	R&D	MAB1195	1:200
KLF17	ATLAS Antibodies	HPA024629	1:500
5-mC	Eurogentec	BI-MECY-0100	1:250
AFP	Abcam	ab169552	1:100
FOXA2	R&D	AF2400	1:100
SMA	Sigma	A2547	1:200
APC-CD172a	Miltenyi Biotec	130-099-785	
PE-anti-human CD106 (VCAM-1)	BioLegend	305806	

Tables S4–S7, related to Figures 2 and S3: RNA-seq expression data (available online).

Supplemental Experimental Procedures

Colony formation assays

1000 HNES or conventional PSCs were seeded in each well of a 12-well plate. Cultures were fixed and stained for alkaline phosphatase at day 7. Images were acquired automatically with the ProScan II system (Prior Scientific) and quantified with the cell counter from ImageJ. For ROCK inhibitor (Y-27632) withdrawal assays, 2×10^3 or 1×10^4 cells were seeded in each well of a 12-well plate in t2iLGö with or without Y-27632 for five days. Cultures were stained for alkaline phosphatase and images acquired with ProScan II.

Cardiomyocyte differentiation

Primed HNES cells were explanted into 6-well plates at approximately 30% confluence. Two to three days later (~70% confluence) differentiation medium was administered following the protocol described previously (van den Berg et al., 2016). APC-CD172a (Miltenyi Biotec, 130-099-785) and PE-anti-human CD106 (VCAM-1) (BioLegend, 130-099-785) were used to identify cardiomyocytes by flow cytometry.

Embryoid body (EB) differentiation

Differentiation medium comprised GMEM (Sigma G5154) with L-glutamine (2 mM), sodium pyruvate (1 mM), 2-mercaptoethanol (0.1 mM), non-essential amino acids (1×, Life Technologies) plus 20% fetal bovine serum for primed PSC or 20% KnockOut Serum Replacement (KSR) for HNES cells. 10 μM ROCK inhibitor was added for the first day of aggregation. Cultures were maintained in 5% O_2 and 7% CO_2 in a humidified incubator at 37°C. QIAshredder (QIAGEN) was used to homogenize EB samples for RNA extraction.

Immunofluorescence staining

Immunostaining on cultured cells and EB outgrowths were performed using standard protocols. Briefly, samples were fixed in 4% PFA and permeabilized with PBS/0.3% Triton-X100. Following one hour of blocking in PBS/0.1% Triton-X100/5% donkey serum, primary antibody staining was carried out in the blocking solution. 5mC staining was performed as described (Ficz et al., 2013). Antibodies used in this study were: OCT3/4 (Santa Cruz sc-5279 (1:200 dilution)), NANOG (eBioscience 14-5769-82 (1:200)), KLF4 (Santa Cruz sc-20691 (1:400)), TFCP2L1 (R&D AF5726 (1:500)), KLF17 (ATLAS Antibodies HPA024629 (1:500)), AFP (Abcam ab169552 (1:100)), FOXA2 (R&D AF2400 (1:100)), SMA (Sigma A2547 (1:200)), 5-mC (Eurogentec BI-MECY-0100 (1:250)).

Karyotype analysis

G-banding karyotype and CGH array analyses were performed at Medical Genetics Laboratories, Cambridge University Hospitals NHS Foundation Trust following their standard protocols. Genomic DNA was isolated with the Qiagen Blood & Tissue kit and profiled on the Affymetrix CytoScan 750K SNP genotyping array. Chromosomal integrity was assessed with the Affymetrix Chromosome Analysis Suite (ChAS) based on human genome build GRCh37/hg19. Data are available in the ArrayExpress repository under accession E-MTAB-4463.

Flow cytometry

Flow cytometry analyses were performed with a Dako Cytomation CyAn ADP high-performance cytometer with Summit software. Data were analyzed using FlowJo.

Reverse transcription and real-time PCR

Total RNA was extracted with the RNAeasy kit (QIAGEN). cDNA was synthesized using SuperScript III (Invitrogen) with oligo-dT adapter primers (GACCACGCGTATCGATGTCGACTTTTTTTTTTTTTTTTT). PCR was performed with the TaqMan assays listed in Supplementary Table 2.

Transcriptome sequencing

Feeder cells were depleted by culture on gelatin-coated dishes for 40 minutes and harvesting cells in suspension. Total RNA was extracted with the TRIzol/chloroform method (Invitrogen), followed by re-suspension in RNAsecure (Ambion), incubation with TURBO DNase (Ambion) at 37ºC for 1 hour, and further phenol/chloroform extraction and ethanol precipitation. RNA integrity was assessed with the RNA 6000 Nano assay on the 2100 Bioanalyzer (Agilent). Ribosomal RNA was depleted from 5 µg of total RNA using Ribo-Zero capture probes (Epicentre). RNA samples were sheared by ultrasonication on a Covaris S2 for 90 s set at Duty Cycle 10, Cycles per Burst 200 and Intensity 5. Fragmented RNA was reverse-transcribed with a combination of random hexamer and oligo-dT primers (New England Biolabs) by SuperScript III (Invitrogen) at 50ºC for 2 hours in the presence of 6 µg/mL actinomycin D (Sigma) to inhibit formation of second-strand products (Perocchi et al., 2007). Second-strand cDNA was synthesized by DNA Polymerase I in the presence of RNase H with dUTPs substituted for dTTPs at 16ºC for 2 hours. Sequential end repair and 30′-adenylation of cDNA products were carried out with T4 DNA polymerase and T4 polynucleotide kinase (20ºC), and with exo^{-} Klenow fragment (65ºC) in the presence of dATPs (New England BioLabs). Products were ligated to barcoded adapters (NEXTflex-96, Bioo Scientific) by T4 DNA ligase (New England BioLabs) at 20ºC for 30 min. Second-strand DNA was digested with uracil DNA glycosylase (UDG) and Endonuclease VIII at 37ºC for 30 min. PCR amplification of first-strand library constructs was carried out with KAPA HiFi DNA polymerase (KAPA Biosystems) for 13 cycles using denaturing and annealing conditions optimized for even A/T versus G/C processing (Aird et al., 2011). Purification of reaction products at each step was performed with Ampure XP paramagnetic beads (Beckman Coulter). Library size distribution and molarity were assessed by DNA 1000 micrifluidic chips on the 2100 Bioanalyzer (Agilent). Sequencing was performed on the Illumina HiSeq 2500 in 125bp paired-end format. Data are available from ArrayExpress and ENA via accession E-MTAB-4461.

RNA-seq data analysis

Additional RNA-seq data from published studies were obtained from the European Nucleotide Archive (ENA (Silvester et al., 2015)). Data were compiled from conventional and reset H9 cells previously described (accession ERP006823, (Takashima et al., 2014)), H1 cells sequenced by the ENCODE Consortium (SRP014320, (Djebali et al., 2012)), H9 cells forming part of a comparative study of pluripotent cells at the Wellcome Trust Sanger Institute (ERP007180), H1, H7, H9 and H14 cells cultured in the Thomson lab (http://scor.chem.wisc.edu (Phanstiel et al., 2011)), WIS2 cells in conventional and modified NHSM/4i conditions (SRP045294, (Irie et al., 2015)) and early human embryo cells and explant PSC cultures profiled by single-cell RNA-seq (SRP011546, (Yan et al., 2013) and SRP055810, (Blakeley et al., 2015)). Residual adapter sequences were removed with Cutadapt (Martin, 2011) and reads were aligned to human genome build GRCh38/hg38 with STAR 2.4.2a (Dobin et al., 2013) using the two-pass method for novel splice detection (Engström et al., 2013). Read alignment was guided by GENCODE v23 (Harrow et al., 2012) human gene annotation from Ensembl release 82 (Cunningham et al., 2015) and splice junction donor/acceptor overlap settings were tailored to the corresponding read length of each dataset. Transcripts were quantified with htseq-count (Anders et al., 2015) based on annotation from Ensembl 82. Duplicate entries (1893 of 60619, primarily Y and snoRNAs) were consolidated to retain the transcript with maximum read coverage as the representative gene product. Libraries were corrected for total read counts using size factors computed by the Bioconductor package DESeq (Anders and Huber, 2010). Principal components were computed by singular value decomposition with the prcomp function in the R stats package from variance-stabilized count data. For PCA with asynchronous single-cell data, cell cycle-associated genes in GO:0007049 (208) were omitted, as were genes that registered zero counts in all single-cell samples compared (24611 of 58726 unique). The remaining 25000 most variable genes were used for PCA. For display of expression data, counts were normalized for gene length to yield FPKM values and scaled to the mean expression over all samples. Heatmaps include genes for which a difference in expression was observed (i.e., scaled expression > 1 or < -1 in at least one sample).

Bisulfite sequencing

Post-bisulfite adapter tagging (PBAT) libraries were prepared as previously described (Smallwood et al., 2014) with some modifications. Feeder cells were depleted by culture on gelatin-coated dishes for 40 minutes and harvesting cells in suspension. Pelleted cells were treated with lysis buffer (10 mM Tris-Cl pH 7.4 and 2% SDS) and 0.5 μl proteinase K followed by incubation at 37°C for 1 hour. Bisulfite conversion was performed on cell lysates using the EZ DNA Methylation-Direct MagPrep Kit (Zymo). After purification, first-strand synthesis was performed using 6N-forward oligos for 37°C for 30 min. Subsequently, samples were treated with Exonuclease I at 37°C for 1 hour, before DNA was purified using 0.8× Agencourt Ampure XP beads (Beckman Coulter). Samples were eluted in second-strand synthesis mix with 6N-reverse oligo and incubated at 37°C for 90 min. DNA was purified using 0.8× Agencourt Ampure XP beads and amplified with KAPA HiFi HotStart DNA Polymerase (KAPA Biosystems) using indexed iPCRTag primers (Quail et al., 2012). Amplified libraries were pooled, purified using 0.8× Agencourt Ampure XP beads, and assessed for quality and quantity using High-Sensitivity DNA chips on the 2100 Bioanalyzer (Agilent) and the KAPA Library Quantification Kit for Illumina (KAPA Biosystems). Libraries were sequenced in 150bp single-end format on the Illumina MiSeq or 125bp paired-end on the Illumina HiSeq 2500. Data are available from ArrayExpress and ENA via accession E-MTAB-4462.

BS-seq data analysis

Sequencing reads were processed to remove the first 6 bases, adapter sequences and poor-quality reads using Trim Galore v0.3.8 (http://www.bioinformatics.babraham.ac.uk/projects/trim_galore, parameters: --clip_r1 6). The remaining sequences were mapped to human genome build GRCh37/hg19 using Bismark v0.13.1 (parameters: --bowtie2 --pbat) (Krueger and Andrews, 2011), and CpG methylation calls were extracted and analysed using SeqMonk (www.bioinformatics.babraham.ac.uk/projects/seqmonk) and custom R scripts. Global comparison of methylation between samples was calculated by averaging CpG methylation levels over 500kb windows.

Oxygen consumption rate (OCR)

OCR was measured using an XF24 Analyzer (Seahorse Bioscience). Seahorse plates were pre-treated with Cell-Tak Cell and Tissue Adhesive (Corning, 4 μg/cm^2) according to the manufacturer's protocol, and 200,000 cells were seeded on each well immediately prior to the experiment. Culture medium was replaced by XF Base Medium (Seahorse Bioscience) supplemented with 2 mM sodium pyruvate and 10 mM glucose with an adjusted pH of 7.4. Cells were then incubated at 37°C in atmospheric CO_2 for one hour. Four compounds from the XF Cell Mito Stress Test kit (Seahorse Bioscience) were injected during the assay to achieve the following final concentrations: oligomycin (1 mM), FCCP (0.5mM), antimycin A (1 mM) and rotenone (1 mM). O_2 consumption was subsequently normalized to the protein content of each well.

Mitochondrial membrane potential

Dissociated cells were plated in growth medium onto Ibidi μ-plates pre-treated with Cell-Tak adhesive reagent (Roche). MitoProbe DiIC1 (5) (Life Technologies) was added at 50 mM and incubated at growth condition for 30 min before imaging (Leica DMI3000). After initial imaging, the uncoupling agent FCCP (0.5 mM) was added and samples were imaged again to determine background staining.

References

Aird, D., Ross, M.G., Chen, W.S., Danielsson, M., Fennell, T., Russ, C., Jaffe, D.B., Nusbaum, C., and Gnirke, A. (2011). Analyzing and minimizing PCR amplification bias in Illumina sequencing libraries. Genome Biol *12*, R18.

Anders, S., and Huber, W. (2010). Differential expression analysis for sequence count data. Genome Biol *11*, R106.

Anders, S., Pyl, P.T., and Huber, W. (2015). HTSeq--a Python framework to work with high-throughput sequencing data. Bioinformatics *31*, 166-169.

Blakeley, P., Fogarty, N.M., Del Valle, I., Wamaitha, S.E., Hu, T.X., Elder, K., Snell, P., Christie, L., Robson, P., and Niakan, K.K. (2015). Defining the three cell lineages of the human blastocyst by single-cell RNA-seq. Development *142*, 3151-3165.

Cunningham, F., Amode, M.R., Barrell, D., Beal, K., Billis, K., Brent, S., Carvalho-Silva, D., Clapham, P., Coates, G., Fitzgerald, S., *et al.* (2015). Ensembl 2015. Nucleic Acids Res *43*, D662-669.

Djebali, S., Davis, C.A., Merkel, A., Dobin, A., Lassmann, T., Mortazavi, A., Tanzer, A., Lagarde, J., Lin, W., Schlesinger, F., *et al.* (2012). Landscape of transcription in human cells. Nature *489*, 101-108.

Dobin, A., Davis, C.A., Schlesinger, F., Drenkow, J., Zaleski, C., Jha, S., Batut, P., Chaisson, M., and Gingeras, T.R. (2013). STAR: ultrafast universal RNA-seq aligner. Bioinformatics *29*, 15-21.

Engström, P.G., Steijger, T., Sipos, B., Grant, G.R., Kahles, A., Ratsch, G., Goldman, N., Hubbard, T.J., Harrow, J., Guigo, R., *et al.* (2013). Systematic evaluation of spliced alignment programs for RNA-seq data. Nat Methods *10*, 1185-1191.

Ficz, G., Hore, T.A., Santos, F., Lee, H.J., Dean, W., Arand, J., Krueger, F., Oxley, D., Paul, Y.L., Walter, J., *et al.* (2013). FGF Signaling Inhibition in ESCs Drives Rapid Genome-wide Demethylation to the Epigenetic Ground State of Pluripotency. Cell Stem Cell *13*, 351-359.

Harrow, J., Frankish, A., Gonzalez, J.M., Tapanari, E., Diekhans, M., Kokocinski, F., Aken, B.L., Barrell, D., Zadissa, A., Searle, S., *et al.* (2012). GENCODE: the reference human genome annotation for The ENCODE Project. Genome Res *22*, 1760-1774.

Irie, N., Weinberger, L., Tang, W.W., Kobayashi, T., Viukov, S., Manor, Y.S., Dietmann, S., Hanna, J.H., and Surani, M.A. (2015). SOX17 is a critical specifier of human primordial germ cell fate. Cell *160*, 253-268.

Krueger, F., and Andrews, S.R. (2011). Bismark: a flexible aligner and methylation caller for Bisulfite-Seq applications. Bioinformatics *27*, 1571-1572.

Martin, M. (2011). Cutadapt removes adapter sequences from high-throughput sequencing reads. EMBnet *17*, 10-12.

Perocchi, F., Xu, Z., Clauder-Munster, S., and Steinmetz, L.M. (2007). Antisense artifacts in transcriptome microarray experiments are resolved by actinomycin D. Nucleic Acids Res *35*, e128.

Phanstiel, D.H., Brumbaugh, J., Wenger, C.D., Tian, S., Probasco, M.D., Bailey, D.J., Swaney, D.L., Tervo, M.A., Bolin, J.M., Ruotti, V., *et al.* (2011). Proteomic and phosphoproteomic comparison of human ES and iPS cells. Nat Methods *8*, 821-827.

Quail, M.A., Otto, T.D., Gu, Y., Harris, S.R., Skelly, T.F., McQuillan, J.A., Swerdlow, H.P., and Oyola, S.O. (2012). Optimal enzymes for amplifying sequencing libraries. Nat Methods *9*, 10-11.

Silvester, N., Alako, B., Amid, C., Cerdeno-Tarraga, A., Cleland, I., Gibson, R., Goodgame, N., Ten Hoopen, P., Kay, S., Leinonen, R., *et al.* (2015). Content discovery and retrieval services at the European Nucleotide Archive. Nucleic Acids Res *43*, D23-29.

Smallwood, S.A., Lee, H.J., Angermueller, C., Krueger, F., Saadeh, H., Peat, J., Andrews, S.R., Stegle, O., Reik, W., and Kelsey, G. (2014). Single-cell genome-wide bisulfite sequencing for assessing epigenetic heterogeneity. Nat Methods *11*, 817-820.

Takashima, Y., Guo, G., Loos, R., Nichols, J., Ficz, G., Krueger, F., Oxley, D., Santos, F., Clarke, J., Mansfield, W., *et al.* (2014). Resetting transcription factor control circuitry toward ground-state pluripotency in human. Cell *158*, 1254-1269.

van den Berg, C.W., Elliott, D.A., Braam, S.R., Mummery, C.L., and Davis, R.P. (2016). Differentiation of Human Pluripotent Stem Cells to Cardiomyocytes Under Defined Conditions. Methods Mol Biol *1353*, 163-180.

Yan, L., Yang, M., Guo, H., Yang, L., Wu, J., Li, R., Liu, P., Lian, Y., Zheng, X., Yan, J., *et al.* (2013). Single-cell RNA-Seq profiling of human preimplantation embryos and embryonic stem cells. Nat Struct Mol Biol *20*, 1131-1139.
